# *Francisella tularensis*: FupA mutation contributes to fluoroquinolone resistance by increasing vesicle secretion and biofilm formation

**DOI:** 10.1080/22221751.2019.1615848

**Published:** 2019-06-04

**Authors:** Claire Siebert, Helena Lindgren, Sabrina Ferré, Corinne Villers, Sandrine Boisset, Julien Perard, Anders Sjöstedt, Max Maurin, Céline Brochier-Armanet, Yohann Couté, Patricia Renesto

**Affiliations:** a TIMC-IMAG UMR 5525 - UGA CNRS, Grenoble, France; b Centre National de Référence des Francisella, Centre Hospitalo-Universitaire Grenoble Alpes, Grenoble, France; c Laboratory for Molecular Infection Medicine Sweden and Department of Clinical Microbiology, Umeå University, Umeå, Sweden; d Université Grenoble Alpes, CEA, Inserm, IRIG-BGE, Grenoble, France; e Université de Caen Normandie, EA4655 U2RM, Caen, France; f Université Grenoble Alpes, CNRS, CEA, BIG-LCBM, Grenoble, France; g Laboratoire de Biométrie et Biologie Évolutive, Université Claude Bernard Lyon 1, CNRS, UMR5558, Villeurbanne, France

**Keywords:** *Francisella*, OMVs, biofilms, antibiotics, fluoroquinolones

## Abstract

*Francisella tularensis* is the causative agent in tularemia for which the high prevalence of treatment failure and relapse is a major concern. Directed-evolution experiments revealed that acquisition of fluoroquinolone (FQ) resistance was linked to factors in addition to mutations in DNA gyrase. Here, using *F. tularensis* live vaccine strain (LVS) as a model, we demonstrated that FupA/B (Fer-Utilization Protein) expression is linked to FQ susceptibility, and that the virulent strain *F. tularensis* subsp. *tularensis* SCHU S4 deleted for the homologous FupA protein exhibited even higher FQ resistance. In addition to an increased FQ minimal inhibitory concentration, LVSΔ*fupA/B* displayed tolerance toward bactericidal compounds including ciprofloxacin and gentamicin. Interestingly, the FupA/B deletion was found to promote increased secretion of outer membrane vesicles (OMVs). Mass spectrometry-based quantitative proteomic characterization of vesicles from LVS and LVS∆*fupA/B* identified 801 proteins, including a subset of 23 proteins exhibiting differential abundance between both strains which may therefore contribute to the reduced antibiotic susceptibility of the FupA/B-deleted strain. We also demonstrated that OMVs are key structural elements of LVSΔ*fupA/B* biofilms providing protection against FQ. These results provide a new basis for understanding and tackling antibiotic resistance and/or persistence of *Francisella* and other pathogenic members of the *Thiotrichales* class.

## Introduction

*Francisella tularensis* is a Gram-negative, facultative intracellular bacterium responsible for tularemia, a re-emerging infectious disease transmitted by ticks, contact with contaminated animals and drinking contaminated water. This highly infectious (<10 bacteria), aerosolizable, and high-mortality rate pathogen is naturally resistant to penicillin and has been classified as a class A bioterrorism agent by the Centers for Disease Control and Prevention (CDC) [1]. The genus *Francisella* comprises the three following species: *F. tularensis*, *F. novicida*, and *F. philomiragia*. *F. tularensis* is further subdivided into the subspecies *tularensis* (Type A) and *holarctica* (Type B), which are the strains primarily responsible for human disease, whereas *F. philomiragia* and *F. novicida* are avirulent in healthy humans. No safe vaccine is currently available, and only a few antibiotic classes – such as fluoroquinolones (FQ) – are effective in treating tularemia. Over the last two decades, overuse of these drugs in clinical practice led to an increased prevalence of multiple species of highly resistant bacteria, including *Francisella* [2]*.* Indeed, treatment failures and relapses are frequently observed in patients with tularemia and pose a serious threat to public health, making the development of new antibiotics a priority [3].

To investigate how drug resistance emerged in *Francisella* spp., directed experimental evolution protocols have been applied to sensitive strains exposed to increasing FQ concentrations [4–6]. These approaches resulted in the emergence of high-level resistant mutants and confirmed that, in *Francisella* spp. as in most Gram-negative bacteria, DNA gyrase is the primary target of FQ [2]. In a previous study [7], using genomic analysis combined with functional DNA supercoiling and cleavage assays, we showed that other mechanisms, independent of GyrA or GyrB mutations also contribute to FQ resistance in *F. novicida*. Based on careful analysis of both microarrays and next-generation sequencing data, a similar conclusion was drawn with the attenuated *F. tularensis* subsp. *holarctica* live vaccine strain (LVS) [5]. Interestingly, in this strain, in addition to *gyrA* (FTL_0533) mutations, 7 out the 11 third-round ciprofloxacin-resistant isolates had at least one single*-*nucleotide polymorphism (SNP) in the gene FTL_0439, which codes for FupA/B (Fer-Utilization Protein). We therefore hypothesized a role for FupA/B in the resistance of *F. tularensis* LVS against FQ. This hypothesis was supported by whole-genome sequencing of highly resistant clones obtained in our lab after 14 selective passages in the presence of ciprofloxacin [4] which also revealed a high proportion of mutations in *fupA/B*. Mutations such as these arise as second-step events after gyrase mutations are acquired, and are accompanied by a moderate – but significant – increase in the minimal inhibitory concentration (MIC) for FQ. Under our experimental conditions, multiple amino acid substitutions were observed at the C-terminus of FupA/B, but 70% of mutants analyzed displayed an insertion resulting in a frameshift and a premature stop codon at the N-terminal region of the protein (unpublished data). The same event was identified in ciprofloxacin-resistant LVS producing a truncated 40-amino acid protein product by Joel A. Bozue and his team (USAMRIID, personal communication).

FupA/B is a 58 kDa protein required for iron uptake and bacterial virulence in *F. tularensis* LVS [8,9]. It is encoded by the *fupA*/*B* gene which results from a fusion associated with a recombinational deletion event between adjacent *fupA* (FTT_0918) and *fupB* (FTT_0919) genes, two paralogs present in *F. tularensis* subsp. *holarctica* FSC200 and close relatives, including *F. tularensis* subsp. *tularensis* SCHU S4 [10]. Together with FTT_0025c (*fslE*), FTT_0267 (*Francisella* metal and virulence; *fmvA*) [11], FTT_0602c (*fmvB*) [11], *fupA* and *fupB, fupA/B* belong to a family of genes encoding outer membrane proteins which are thought to be unique to the *Francisella* genus [12]. FupA/B contains the first 297 amino-terminal residues of FupA and the C-terminal region of FupB (Supp Fig. 1). Whereas decreased virulence accompanies *fupA* and *fupB* gene fusion in *F. tularensis* SCHU S4 [8,9], the chimeric protein retains the high-affinity ferrous iron transport capability of FupA [13]. Furthermore, unlike the FupA protein from the SCHU S4 strain, FupA/B can also mediate siderophore-dependent ferric iron uptake [13]. Whether the hypothetical link between *fupA/B* mutations and ciprofloxacin susceptibility [5] is another specific feature of the fusion gene, or whether it is inherited from *fupA* and/or *fupB* ancestors is currently unknown. In agreement with the latter hypothesis, we observed that a highly FQ-resistant mutant of *F. novicida* (U112_Fno3) bearing a functionally-inert DNA gyrase mutation (GyrA_ΔE524, ΔS525) also had a nonsense mutation in FTN_0444 (*fupA*) [7]. Moreover, *fupA* was identified as one of seven mutated genes in a ciprofloxacin-resistant mutant of *F. tularensis* SCHU S4, alongside *gyrA* and *parE* [14].

In the present study, we investigated whether *fupA/B* deletion is responsible for FQ stress resistance in *Francisella* spp through trans-complementation of *F. tularensis* LVS deletion mutants and we assessed the consequences of its knockout. Among the phenotypic changes associated with FupA/B deletion are increased secretion of outer membrane vesicles (OMVs) and formation of biofilms that provide bacteria with better survival opportunities through enhanced resistance toward antibiotic stresses. This work provides new insights into the mechanisms behind *F. tularensis* resistance/tolerance toward FQ, which could be useful when developing new therapeutic strategies.

## Materials and methods

### Bacterial strains and growth conditions

*F. tularensis* subsp *holarctica* LVS (NCTC 10857) and *F. tularensis* subsp *tularensis* SCHU S4 were used as parental strains. LVS strains were grown on Polyvitex-enriched chocolate agar (PVX-CHA) (bioMérieux, Marcy L'Etoile, France) and SCHU S4 on Modified Mueller-Hinton (MMH) agar plates incubated at 37°C in a 5% CO_2_-enriched atmosphere for 48–72 h. Liquid cultures were carried out at 37°C in different media, as indicated. All experiments were conducted in a biosafety level 3 laboratory. LVSΔFTL_0439 (LVSΔ*fupA/B*) was produced by a two-step process and using the suicide plasmid pMP812 as previously described [15,16].

### Tolerance measurement

The tolerance was first determined by the Minimal Bactericidal Concentration/Minimal Inhibitory Concentration (MBC/MIC) ratio measured from the 96-well plates used for the MIC determination. Briefly, supernatants were aspirated from wells where no visible bacterial growth was observed and collected samples were diluted before plating onto PVX-CHA plates and incubated at 37°C.

The tolerance was also evaluated using the method based on Minimum Duration for Killing 99% of the population -MDK metric- [17,18] as follows. Bacterial cultures were grown in 10 mL of MMH at 37°C under shaking to OD_600nm_ 0.5 and were challenged with 25 times the MIC of ciprofloxacin (1.6 µg/mL), gentamicin (6.25 µg/mL) or doxycycline (6.25 µg/mL) for 6 h at 37°C under shaking. Every hour, 50 µL of culture were collected and 10-fold serial dilutions were performed in PBS before plating onto PVX-CHA plates. The number of tolerant cells was assessed through CFU counting and data were expressed as a percent of CFU relative to untreated bacteria.

### Isolation and quantification of OMVs

Bacterial cultures were grown in MMH at 37°C under shaking to late logarithmic (OD_600nm_ 0.8) or stationary (OD_600mm_ 2) growth phase. After addition of sodium azide (0.01% w/v), bacteria were pelleted by centrifugation (7,000 × *g*, 10 min) and the supernatants passed through a 0.22-μm filter unit to remove intact cells. In preliminary experiments, recovered supernatants were plated on PVX-CHA plates to ensure sterility. Fourteen ml of filtered supernatants corresponding to 99.4 ± 13.9 × 10^9^ and 105.1 ± 10.45 × 10^9^ stationary phase bacteria for LVS and LVSΔ*fupA/B,* respectively (mean ± SEM; *n* = 3; *P *> 0.05), were ultracentrifuged for 2 h (Sorvall Ultracentrifuge; 100,000 × *g*). The pelleted OMVs were resuspended in 7 ml PBS for washing. The ultracentrifugation step was repeated and the OMVs freshly resuspended in 80 µl PBS for each bacterial strain were used for direct quantification (Nanosight, Malvern Instrument) as well as for SDS-PAGE analysis. When added to bacteria for biofilm formation purpose, sodium azide was omitted in the purification process. The yields of purified vesicles per stationary phase bacteria were of 0.432 ± 0.07 for LVS and of 1.801 ± 0.16 for LVSΔ*fupA/B* (mean ± SEM; *n* = 3; *P *< 0.005).

### Biofilm assay

Biofilm formation was quantified by Crystal violet assay as previously described [19]. Briefly, overnight bacterial cultures grown in MMH at 37°C under shaking were diluted to a concentration of 1.10^9^ cells/mL and 200 µL of the bacterial suspension was added to a flat-bottom 96-well plate. After 72 h at 37°C in a 5% CO_2_ incubator without shaking the OD_600nm_ was measured to normalize bacterial growth (Tecan Plate reader) and bacteria were gently aspirated before three well washings with sterile PBS. The plate was then incubated for 1 h at 70°C, stained with 200 µL of 0.1% (w/v) Crystal violet/well for 15 min and washed as described above. Solubilization and quantification of the biofilms was achieved by addition of 200 µL acetic acid (30% v/v) and measurement of OD at 595 nm. In some experiments, OMVs freshly purified from stationary phase LVSΔ*fupA/B* which is more prone to secrete vesicles, and quantified using the Nanosight Instrument, were added in the wells of the microtiter plate filled with 2 × 10^8^ bacteria. Different ratios of bacteria:OMVs were tested ranging from 1:1–1:20. These ratios were not arbitrary chosen but based on confocal laser-scanning imaging of biofilm formed by LVSΔ*fupA/B* (Figure 6(e)) as well as from the yield of OMV purification per bacteria (see above).

### Evaluation of antimicrobial resistance in biofilms

After two washes of LVSΔ*fupA/B* biofilm with 200 µL PBS, the MMH was replaced with fresh medium supplemented with increasing concentrations of ciprofloxacin (1x to 200x MIC) and the biofilms were incubated at 37°C for 24 h before evaluation of the bacterial viability by both CFU counting and resazurin assay. Upon dilution, the planktonic bacteria harvested from the same wells where biofilms were formed were transferred in another 96-well plate containing ciprofloxacin and processed in parallel.

### Measurement of bacterial viability

Viability was determined using resazurin (7-hydroxy-3H-phenoxazin-3-one 10-oxide; Sigma-Aldrich), a fluorescent indicator of mitochondrial function, which triggers the reduction by viable bacteria of the blue resazurin dye virtually non fluorescent (max absorbance *λ* = 600 nm) to the pink fluorescent compound resorufin (max absorbance *λ* = 570 nm). The biofilms previously exposed or not to antibiotics were gently washed twice with PBS to remove planktonic cells, and 200 μL of a 0.02 mg/mL resazurin (Sigma-Aldrich) solution in MMH was added to each well. After a 2 h incubation at 37°C under static conditions, the absorbance was measured using the Tecan Plate reader to estimate the cell viability (OD_570nm_–OD_600nm_) for each well. For planktonic cells (200 µL), the reaction was initiated by addition of 20 µL of resazurin (0.2 mg/mL) and the bacterial viability was evaluated after 1 h incubation at 37°C. Data are expressed as percent cell viability of untreated cells.

In some experiments, viability of bacteria within biofilms was also evaluated using the membrane impermeant dye propidium iodide (PI, Molecular Probes) that is excluded from viable cells. Biofilms exposed to ciprofloxacin (MIC x2.5 or 0.16 µg/mL, 24 h) or H_2_O_2_ (100 mM, 30 min) used as positive control were incubated with 3 µM PI for 30 min in the dark at room temperature. After gentle washes with PBS, the fluorescence intensities were measured using a microplate reader (excitation 493 nm/ emission 632 nm). In some wells, and before staining, biofilms were fixed with 4% paraformaldehyde for 30 min in order to permeabilize the outer membrane of bacteria and to determine intensity of fluorescence corresponding to a mortality rate of 100%. Results were expressed as the ratio between the fluorescence values measured for biofilms exposed to various treatments and fluorescence values obtained with fixed samples.

### Statistical analysis

All data correspond to biological replicates. Otherwise indicated they were analyzed with Student’s t- tests and using the GraphPad PRISM software. The number of independent data points and *P* values are reported in figure legends.

Experimental details concerning bioinformatics analysis, construction of the FTL_0439 knock-out strain and the FupA/B complementation plasmid, production of anti-FupA antibody, MIC and iron measurements, western-blots, Dynamic Light Scattering, Mass spectrometry-based quantitative proteomic analyses, Quantitative Real-Time PCR, colony forming unit (CFU) counting and fluorescence microscopy are described in Supplementary information.

## Results

### DUF3573-containing proteins are widespread in *Thiotrichales*

**Figure 1.**
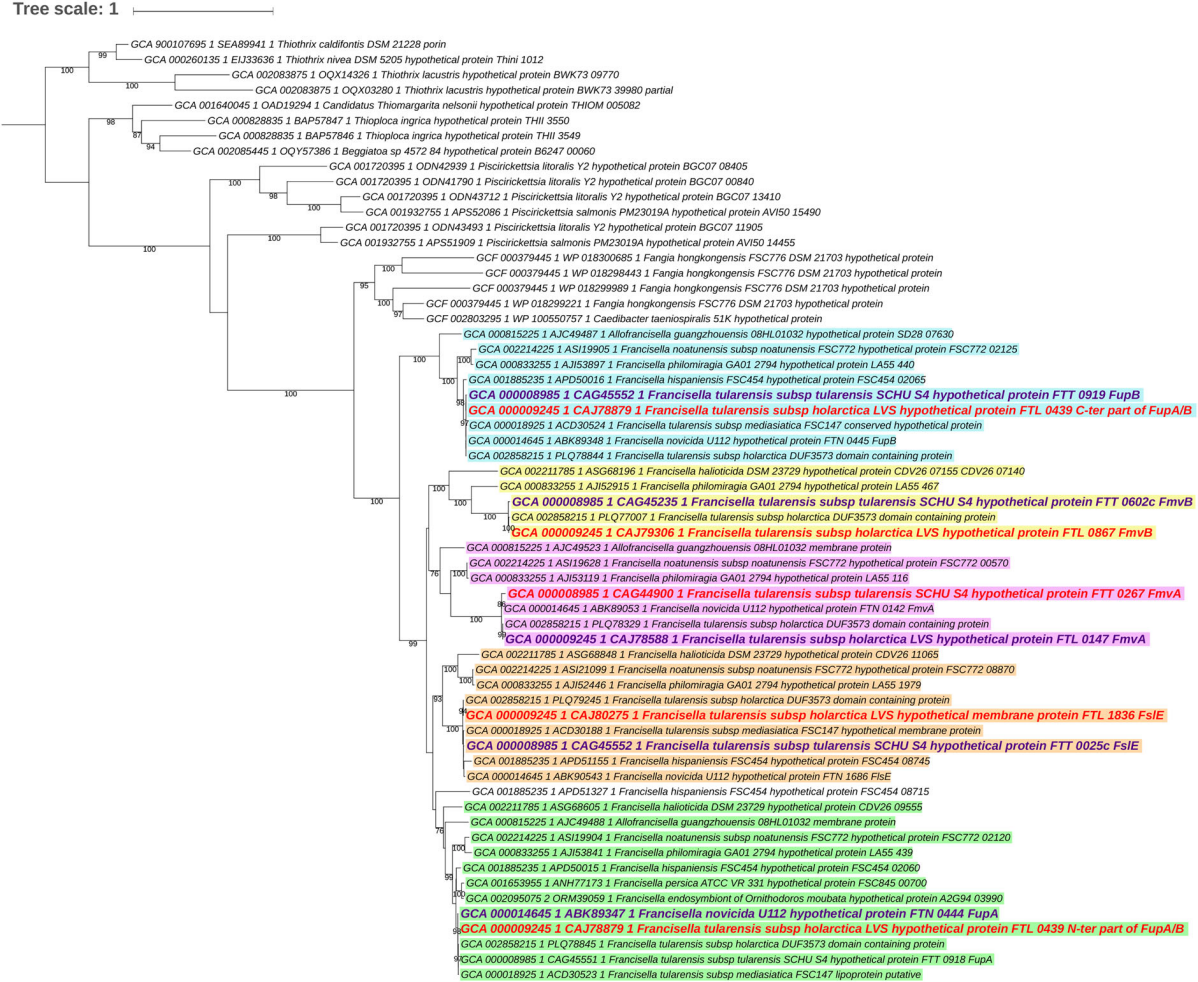
Maximum likelihood tree of DUF3573-containing proteins in *Thiotrichales* (62 sequences, 303 amino acid positions used). The 62 sequences were selected among the 1,214 homologs identified in the 384 *Thiotrichales* proteomes available at the NCBI, by keeping sequences from one representative strain per species or subspecies in the case of *Francisella*. The scale bar represents the average number of substitutions per site. Numbers at nodes correspond to ultrafast approximated bootstra*p* values. For clarity, values lower than 75% are not shown. The five protein subfamilies corresponding to FupA, FupB, FslE, FmvA, and FmvB are shown in green, blue, orange, pink, and yellow. The five sequences from *Francisella tularens*is subsp. *tularensis* SCHU S4 and the four sequences from *F. tularensis* subsp. *holarctica* LVS are shown in purple and red, respectively. Noteworthy, in the latter as in other LVS strains, FupA and FupB are fused. Regions corresponding to FupA and FupB in the LVS strain were analyzed separately to infer this tree. As expected, they group within subfamilies corresponding to FupA and FupB, respectively.

FupA, FupB, and FupA/B all contain a protein domain of unknown function DUF3573 (Supp Table 1). A survey of complete proteomes available in public databases disclosed 1,214 homologs exclusively belonging to members of the *Thiotrichales* order (*Gammaproteobacteria*). More precisely, in addition to *Francisella*, homologs were found in members of *Allofrancisella*, *Beggiatoa*, *Caedibacter*, “*Candidatus* Thiomargarita”, *Fangia*, *Piscirickettsia*, *Thioploca*, and *Thiotrix* genera, but not in *Sulfurivirga*, *Thiomicrospira*, *Hydrogenovibrio*, *Cycloclasticus*, and *Methylophaga* (Figure 1 and Supp Dataset 1). Thus, the taxonomic distribution of FupA/FupB homologs is much broader than previously supposed, as it was assumed to be unique to *Francisella* [12].

The maximum likelihood phylogenetic analysis of the FupA/FupB homologs indicated that the protein family can be divided into five subfamilies corresponding to FupA, FupB, FslE, FmvA and FmvB, each of which contains a DUF3573 domain (Supp Table 1). As expected, the N-ter and the C-ter regions of FupA/B branch within the FupA and FupB subfamilies, respectively. All five subfamilies emerged during the diversification of *Francisella* from a monogenic ancestral family that originated in *Thiotrichales*. According to the inferred maximum likelihood tree, FupB was the first subfamily to diverge (approximated bootstra*p* values (aBV) = 99%); the relative order of emergence of the other four families cannot be confidently determined (aBV < 75%).

Interestingly, the five subfamilies bear distinguishing features that might reflect functional differences (Supp Table 1). In particular, a signal peptide is found in the FupA subfamily (including FupA/B) and in some representatives of the FmvB subfamily, whereas the FupB, FmvA, and FslE subfamilies have a single transmembrane domain located in their very N-ter region. A membrane lipoprotein lipid attachment site is present in FupA (and FupA/B) at position C18, whereas a cysteine protease inhibitor signature is present at position AA 506–519 of FupA proteins (Supp Fig. 1). The functions of these proteins are still very poorly known. FslE was described as a siderophore receptor that shares the remarkable capacity to mediate iron uptake and to be a virulence factor with FupA [20–22]; FmvB was shown to be another virulence factor involved in magnesium, but not iron, uptake [11]. To date, no functional role has been attributed to FupB or FmvA, which are not involved in metal acquisition or in bacterial virulence [11,21,22]. It was recently shown that FupA expression is not regulated by the ferric uptake regulator (Fur) protein and that its expression is independent of iron concentration, leading to the assumption that FupA may have alternative functions in addition to iron transport [23]. Thus, the precise roles of these DUF3573-containing proteins, for which no structural data is available, remain to be clarified.

### FupA/B deletion confers FQ resistance on *F. tularensis* LVS

**Figure 2.**
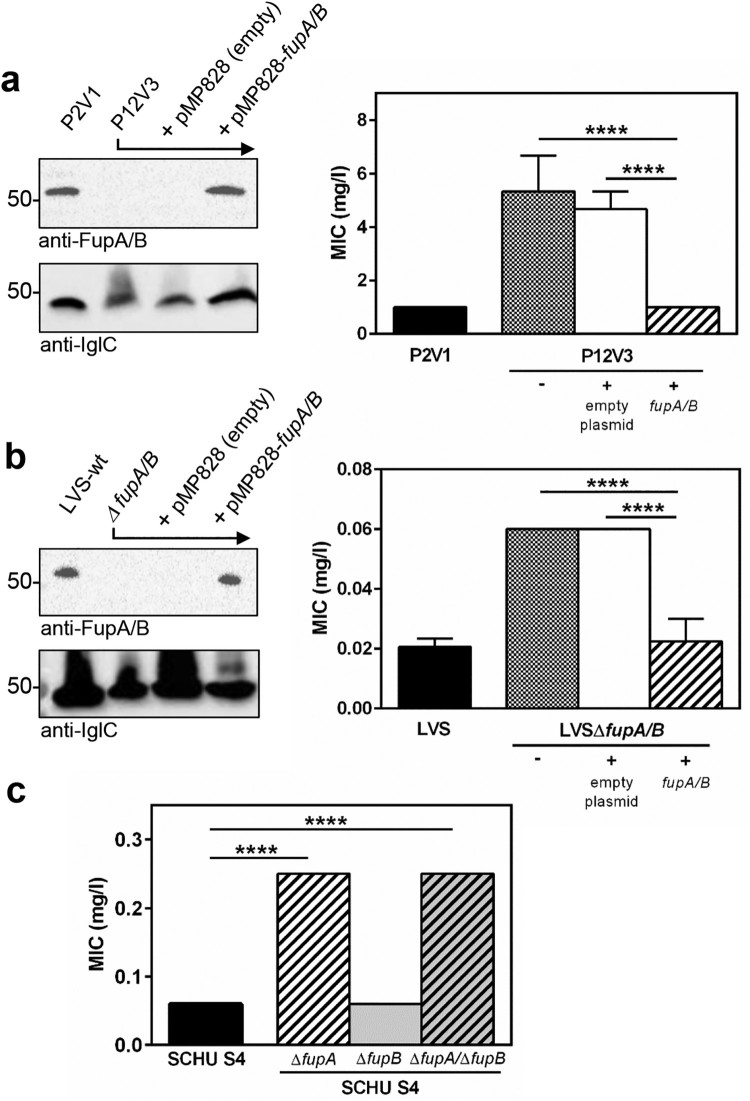
Restoration of ciprofloxacin resistance phenotype in *fupA/B*-complemented mutants. (a) Left panel: FupA/B expression was evaluated by western-blot and using anti-IglC as positive control on whole lysates of LVS mutants resulting from directed-evolution experiments [4] showing that protein expression in P12V3 is restored upon gene trans-complementation using the plasmid pMP828. Right panel: The same strains were assayed for ciprofloxacin susceptibility, measured as MIC values. (b) Same experiments performed on LVS and LVSΔ*fupA/B* strains. (c) Ciprofloxacin-susceptibility of *F. tularensis* SCHU S4 as well as Δ*fupA*, Δ*fupB* and the double Δ*fupA*/Δ*fupB* deletion mutants. MIC were determined from triplicate cultures from three different experiments. **** *P *< 0.0001.

To investigate the impact of FupA/B deletion on FQ resistance in *F. tularensis* LVS, we first complemented resistant mutants. To do so, we took advantage of a directed experimental evolution protocol developed in our lab which involves sub-culturing bacteria in the presence of progressively increasing concentrations of FQ [4]. Because FupA/B mutations arise as a second-step event after gyrase substitutions have been acquired, we included a control: the P2V1 mutant isolated at passage 2, which has a T83 K substitution in GyrA causing (50-fold) lower sensitivity to ciprofloxacin than wild-type LVS (minimal inhibitory concentration (MIC) 1 mg/L *vs.* 0.02 mg/L). The P12V3 mutant, isolated at passage 12, bore the same T83 K substitution as P2V1 along with a nucleotide insertion at position 108 in the FTL_0439 gene, producing a stop codon at position 41 of FupA/B. This mutant also presented a four-amino-acid deletion in the FTL_0594 gene encoding the UDP-glc-4-epimerase, and is significantly more resistant to ciprofloxacin than P2V1 (Figure 2(a)). Importantly, complementation of this clone with the wild-type *fupA/B* not only restored protein expression, but also ciprofloxacin resistance at a level similar to that of the P2V1 parental strain; no difference was observed when an empty plasmid was used (Figure 2(a)). This effect is therefore likely to be specific for FQ since no difference was observed for the other compounds tested, which included doxycycline, azitromycin, gentamicin and linezolid (not shown).

To conclusively demonstrate that FupA/B deletion contributes to FQ resistance and exclude a possible role for the UDP-glc-4 epimerase, we generated a FTL_0439 knock-out mutant of *F. tularensis* LVS (LVSΔ*fupA/B*) (Supp Fig. 2 a-d). In line with the role described for FupA/B in iron acquisition [8,9], LVSΔ*fupA/B* exhibited a lower iron content than the wild-type strain, a defect compensated for by trans-functional complementation of *fupA/B* (Supp Fig. 2e). As shown in Figure 2(b), deletion of *fupA/B* in LVS increased the ciprofloxacin MIC 3-fold, whereas a sensitivity comparable to the wild-type was restored in the rescue strain. Although the ciprofloxacin MIC values for these strains – which lack DNA gyrase mutations – were much lower than those of the directed-evolution mutants, these results clearly confirm the role played by FupA/B in FQ sensitivity. In agreement with the observations from the directed-evolution strains, this effect was specific for FQ (not shown).

To investigate whether the functional link between FupA/B and FQ resistance was inherited from FupA and/or FupB, or whether this effect was specific to the fusion protein, we compared the ciprofloxacin-susceptibility of the highly virulent *F. tularensis* SCHU S4 to that of mutants lacking *fupA, fupB* or both. The results obtained showed that FupA, but not FupB deletion leads to increased FQ resistance in *F. tularensis* SCHU S4 (Figure 2(c)). Remarkably, and in line with the link between FupA expression and the phenotypic FQ sensitivity, the SCHU S4 mutant harboring the Δ*fupA*/Δ*fupB* double deletion had a susceptibility-profile similar to that of SCHU S4Δ*fupA*.

### FupA/B deletion generates a phenotype of tolerance to bactericidal antibiotics

**Figure 3.**
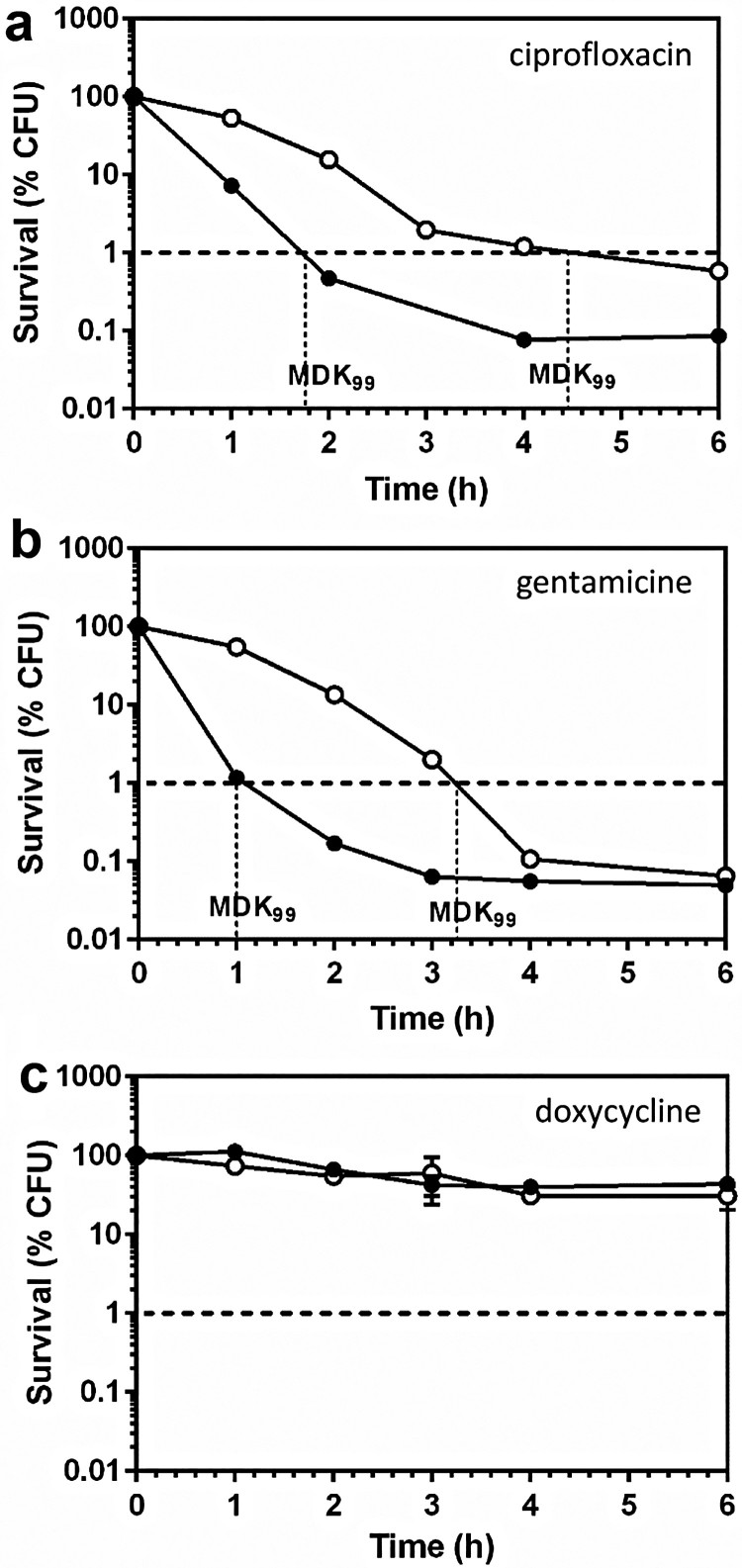
Killing curves of LVS and LVSΔ*fupA/B.* Exponential growth phase LVS (black circles) or LVSΔ*fupA/B* (white circles) were exposed to 25x the MIC of (a) ciprofloxacin (0.5 and 1.6 mg/L respectively), (b) gentamicin (6.25 mg/L) or (c) doxycycline (6.25 mg/L) and the CFU were determined by plating. This graph is representative of 3 independent experiments.

Tolerant and non-tolerant bacteria may not be distinguishable when considering MIC values alone, therefore, we next investigated whether the FupA/B deletion contributed to FQ tolerance in *Francisella.* For this part of our study, we first determined the minimal bactericidal concentration (MBC)/MIC ratios which were found to be of 66 *vs* 8.3 for LVSΔ*fupA/B* and the wild-type strain, respectively (*n* = 3). The Clinical Laboratory Standards Institute (CLSI) guidelines define tolerance as a MBC/MIC ratio >32, thus LVSΔ*fupA/B* effectively exhibits a tolerant phenotype [24]. This result was confirmed by estimation of the MDK_99_ (Minimum Duration for Killing 99% of the population), which is a recently proposed alternative measure of tolerance [17,18]. As illustrated in Figure 3(a), when bacteria were exposed to ciprofloxacin at 25x its MIC, the time required to reach 99% killing was substantially longer for LVSΔ*fupA/B* than for the wild-type strain. This increase in survival of the FupA/B-deleted strain was also observed with gentamicin (Figure 3(b)), while exposure of bacteria to doxycycline at 25x its MIC failed to induce 99% killing within the same time period (Figure 3(c)).

### *F. tularensis* LVS lacking FupA/B produce more OMVs

**Figure 4.**
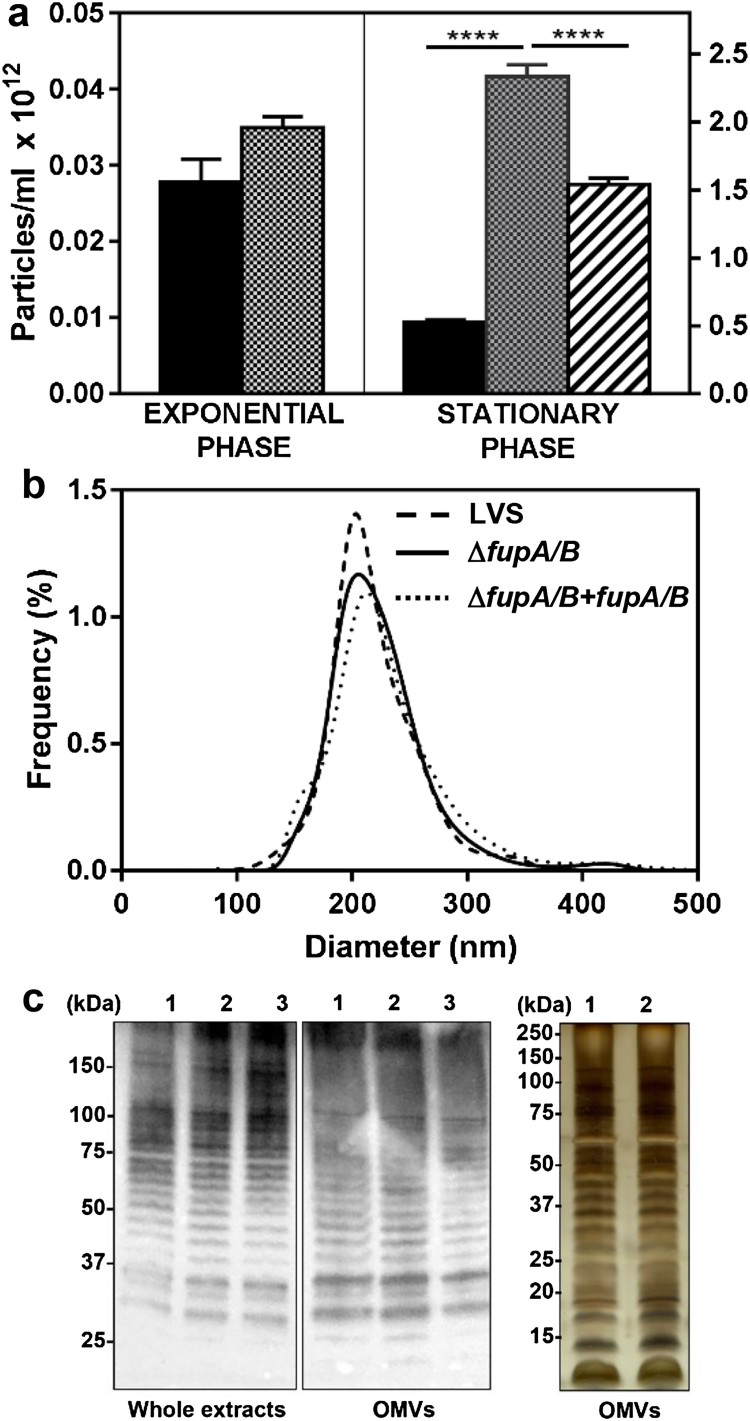
Quantitative and qualitative analysis of OMVs in *F. tularensis* LVS. (a) Nanosight enumeration of OMVs purified from *F. tularensis* LVS (black columns), Δ*fupA/B* mutant (dotted columns) and Δ*fupA/B* mutant complemented with *fupA/B* (hatched columns). Bacteria grown in MHM at 37°C under shaking were collected either at the exponential or stationary growth phase, as indicated. Because no significant difference was shown between bacteria in exponential phase of growth, the complemented mutant was not considered under such conditions. Data are expressed as mean ± SEM of 4 different experiments. ****P < 0.0001. (b) Nanosight sizing of OMVs purified from stationary growth phase bacteria. (c) LPS and protein profiles of whole bacterial lysates and of purified OMVs. Left and middle panels: whole bacterial extracts (5 µg) and OMVs samples (1.5 µg) from (1) LVS, (2) Δ*fupA/B* or (3) Δ*fupA/B + fupA/B* strains were separated on 4-20% gradient gel before transfer and immunostaining with the anti-LPS antibody. Right panel: Silver staining of OMV proteins from (1) LVS, (2) Δ*fupA/B* (1.5 µg) separated on 12% SDS-PAGE.

Bioinformatics analysis of the DUF3573-containing protein family (Supp Fig. 1, Supp Table 1) revealed FupA and FupA/B to share a lipoprotein signature in their N-ter region. One of the functional roles of lipoproteins is to ensure envelope stability of Gram-negative bacteria [25]. Consequently, deletion of FupA/B may lead to outer membrane instability, and indeed, this hypothesis is supported by experiments in *F. novicida* U112 showing the presence of a greater abundance of proteins in the culture supernatant with a Δ*fupA* (FTN_0444) strain compared to the wild-type [26]. In agreement with these observations with *F. novicida* U112*,* a comparative proteomics analysis of LVS and LVSΔ*fupA/B* secretomes revealed strong enrichment of several proteins in the LVSΔ*fupA/B* supernatant (not shown). LDH-release assays indicated that this increase in proteins was unrelated to bacterial lysis, but the proteins identified included an unexpectedly large proportion of cytosolic proteins. In several Gram-negative bacteria, deletion of lipoproteins was found to promote OMV biogenesis [27]. This finding, together with the increased number of surface protrusions observed on LVS lacking *fmvB*, another member of the DUF3573-containing protein family [11], prompted us to explore the influence of FupA/B deletion on OMV production. Particle counts and sizes compared using a Nanoparticle Tracking instrument (Supp Movie 1) revealed that *fupA/B* deletion induces an approximately 5-fold increase in the number of OMVs produced at the stationary phase (Figure 4(a)). This hypervesiculating phenotype is reversed upon complementation with FupA/B. The OMV size-distribution for the different strains was similar, with mean diameters of around 220 nm (Figure 4(b)). This result was confirmed by dynamic light scattering (DLS) which estimates a hydrodynamic diameter of 250.9 ± 2.96 and 249.9 ± 2.79 nm for LVS and LVSΔ*fupA/B* OMVs, respectively (mean ± SEM; *n* = 3; *P* > 0.05). Immunostaining of whole bacterial lysates and OMVs with anti-LPS antibody showed similar ladder-like patterns for the three strains (Figure 4(c), left and middle panels). Interestingly, LPS profiles from bacteria and OMVs were quite similar as was the protein content in OMVs produced by wild-type LVS and Δ*fupA/B* mutants (as revealed by silver-stained SDS-PAGE; Figure 4(c), right panel).

### Subtle difference in OMV proteomes from wild-type *F. tularensis* LVS and Δ*fupA/B*

**Figure 5.**
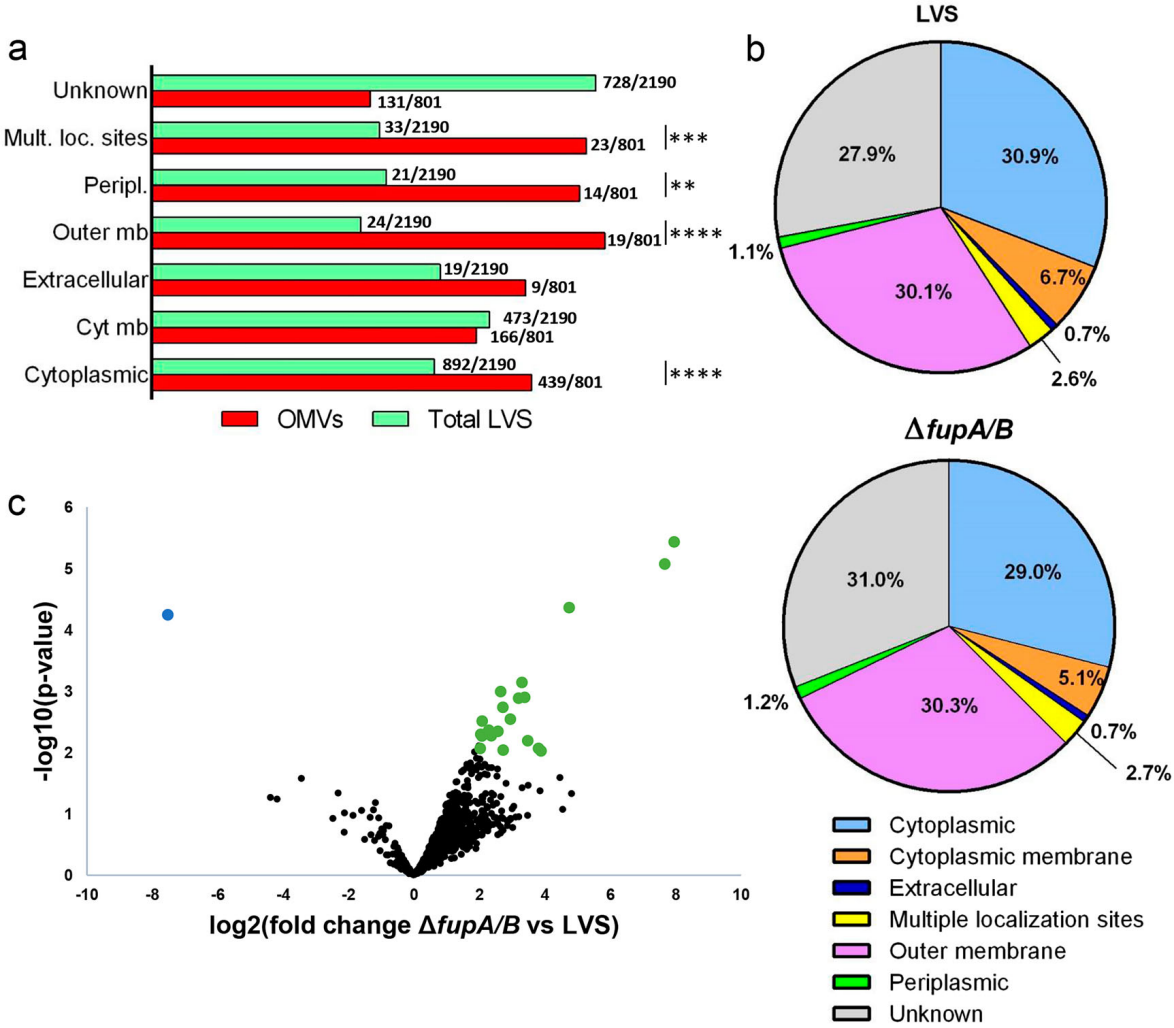
Proteomic characterization of OMV proteomes from wild-type *F. tularensis* LVS and the Δ*fupA/B* mutant. (a) Localization of proteins from total proteome (2190 proteins) and purified OMVs (801 identified proteins) as predicted by PSORTb. Over-representation of OMVs proteins compared to total proteome in each category was tested using Fisher’s exact test (**P <* *0.01, ***P <* *0.001, ****P <* *0.0001). (b) Predicted localization of proteins identified in OMVs from wild-type LVS strain and Δ*fupA/B* mutant. Each category is represented by the summed abundances (iBAQ values) of the contributing proteins. (c) Volcano plot representing the -log_10_(*P*-value) plotted against the log_2_(fold change) for each quantified OMV protein. One protein from the mutant strain (blue dot) and twenty-two proteins from the wild-type strain (green dots) were found to be statistically differentially enriched.

To finely characterize the protein content of OMVs from wild-type *F. tularensis* LVS and its Δ*fupA/B* mutant, we undertook an MS-based quantitative proteomic analysis. Bioinformatic analyses and statistical filtering allowed us to identify 801 different proteins with high confidence, which were detected in at least three biological replicates for one condition (Supp Table 2; ProteomeXchange dataset PXD010305). Because the protocol applied to enrich OMVs did not include a density gradient ultracentrifugation step, we cannot exclude a partial contamination of OMVs with residual exogenous proteins such as filamentous appendages or large protein complexes. However, we noticed that the majority of these 801 proteins were already identified in published proteomics characterizations of membrane fractions prepared from *F. tularensis* LVS strain [28,29] (Supp Table 2). Interestingly, we identified the FtlA lipase recently shown to be a component of *F. tularensis* LVS OMVs [30], as well as 13 proteins encoded by the *Francisella* pathogenicity island locus 1 and 2 (FTL_0111 to FTL_0126 and FTL_1157 to FTL_1172). Using LipoP [31], lipoprotein signal peptides were predicted for 53 of the proteins identified. Comparison of the PSORTb-predicted localizations [32] of the *in silico*-translated total LVS proteome with our 801-protein repertoire indicated that the OMV fraction is enriched in outer membrane and periplasmic proteins, as expected, but also contains cytoplasmic proteins, or proteins with multiple localizations (Figure 5(a)). By crossing intensity-based absolute quantification (iBAQ) data with localization prediction for each protein identified, the majority of the total protein amount in the OMV fraction corresponded to outer membrane proteins, cytoplasmic proteins and proteins of unknown localization. A similar localization profile was obtained whether OMVs were prepared from wild-type *F. tularensis* LVS or the Δ*fupA/B* mutant (Figure 5(b)). However, a thorough quantitative comparison of the proteomes of OMVs purified from both strains revealed 23 proteins, including FupA/B, with statistically significant differential abundance (Figure 5(c), Table 1). Among these proteins, all but FupA/B were found to be enriched in OMVs purified from the *F. tularensis* LVSΔ*fupA/B* mutant. Interestingly, this group of 22 overexpressed proteins was found to be statistically enriched in predicted lipoproteins (Fisher’s exact test *P*-value <0.02).

**Table 1.** Proteins whose abundance significantly differs in FupA/B-deleted strain as compared with wild-type LVS.

ORF	Description	Proteomic data^a^			RT-qPCR data^b^
		Score	*P*-Value	Log2FC	Log2FC
FTL_0439	DUF3573 domain-containing protein FupA/B	196.48	5,5226E-05	−7,52577196	nd
FTL_1931	Hypoxanthine-guanine phosphoribosyl transferase 2.4.2.8	35.762	3,7272E-06	7,95033342	4,97 ± 0,24
FTL_1836	DUF3573 domain-containing protein	106.53	8,1905E-06	7,66110499	4,45 ± 1,26
FTL_1835	Bcr/CflA family drug resistance efflux transporter	10.788	4,2138E-05	4,74375095	2,45 ± 0,50
FTL_1842	Aspartyl/glutamyl-tRNA amidotransferase subunit A	25.759	0,00070266	3,29921922	3,5 ± 0,37
FTL_0146	ABC transporter, ATP-binding protein	66.127	0,00103677	2,64293944	1,89 ± 0,38
FTL_0012	Recombinase A	23.,629	0,00127529	3,38036766	1,86 ± 0,48
FTL_1644	Glycerol kinase	1.5761	0,001313	3,18959478	0,89 ± 0,33
FRATH_1496	Conserved exported protein of unknown function	7.4103	0,0018331	2,71240949	nd
FTL_1850	Adenylosuccinate lyase 4.3.2.2	76,329	0,00290404	2,93821586	1,47 ± 0,10
FTL_1274	Biotin synthesis protein BioC	8,5509	0,00310019	2,08179398	4,86 ± 1,39
FTL_1968	Ribonuclease G	1.8003	0,0043569	2,28108698	nd
FTL_1365	Hypothetical protein	10.001	0,00444144	2,55844422	nd
FTL_0203	VWA domain-containing protein	67,725	0,00496868	2,28589582	6,56 ± 0,64
FTL_1724	Outer membrane protein assembly factor BamB	188,45	0,0050169	2,19810953	6,78 ± 1,06
FTL_1025	30S ribosomal protein S18	2,9555	0,00502734	2,03769224	2,6 ± 0,50
FTL_1045	Conserved hypothetical lipoprotein	50,354	0,00524306	2,362267	2,12 ± 0,36
FTL_1407	Threonyl-tRNA synthetase	20,082	0,00553302	2,07539867	1,73 ± 0,31
FTL_0449	HTH-MerR Superfamily transcription regulator protein	10,91	0,00624757	3,4734326	0,96 ± 0,46
FTL_1591	Acetyl-CoA carboxylase, biotin carboxylase subunit	73,596	0,00860376	2,01843356	2,38 ± 0,85
FTL_1899	Glutamine synthetase 6.3.1.2	74,386	0,00862336	3,80186705	1,23 ± 0,44
FTL_0738	Hypothetical lipoprotein	53,597	0,00923321	3,8760831	2,29 ± 1,29
FTL_1832	lucA/IucC family siderophore biosynthesis protein	16,088	0,00933872	2,72099163	3,9 ± 1,016

Changes in protein expression in Δ*fupA/B* compared to LVS found with proteomic analysis were expressed as Log2 (Fold Change) (Log2FC). With the exception of FupA/B (FTL_0439), all identified proteins were overexpressed in Δ*fupA/B.* These results were confirmed by RT-qPCR performed on at least two different batches of total RNA extracted from 24 h bacterial cultures and using 16SrRNA as control. Only transcripts from proteins identified by at least two peptides were retained for RT-qPCR.

^a^See details in Supp Table 2.

^b^Values represent the average ± SEM of 3 to 6 measurements. (n.d.) not determined.

Strikingly, among the 22 proteins enriched in OMVs purified from the Δ*fupA/B* mutant, FTL_1836, corresponding to the paralogous FupA protein FslE (Supp Table 1), was one of the proteins with the highest level of overexpression. Strains deleted for *fupA/B* also produced vesicles with increased amounts of proteins playing a role in iron uptake, such as proteins involved in the synthesis of the lucA/lucC siderophore family (FTL_1832). The LVSΔ*fupA/B* OMVs also contained more proteins playing a role in replication (FTL_1025, FTL_1407, FTL_1842, FTL_1850, and FTL_1931), glycerolipid (FTL_1644), carbohydrate (FTL_1591 and FTL_1899), and cofactor and vitamin (FTL_1274) metabolism. Some of the differentially-expressed proteins identified may play a direct role in the antibiotic-resistance phenotype of the strain. They include membrane transport proteins that could contribute to cellular permeability to drugs, such as the Bcr/CflA drug resistance efflux transporter (FTL_1835), the ABC transporter ATP-binding protein (FTL_0146), and the outer membrane protein assembly factor BamB (FTL_1724). Among the proteins upregulated in LVSΔ*fupA/B* is RecA (FTL_0012), a key activator of the widespread SOS response which allows bacteria to counteract DNA damage and promotes survival following FQ exposure [33,34]. The SOS response can also be induced by several other antibiotics including aminoglycosides [34], therefore, upregulation of RecA could also affect the outcome of gentamicin exposure, leading to the tolerance phenotype observed.

To check whether the upregulation observed were simply the result of selective packing of proteins during OMV biogenesis [35,36] or whether they reflect differential expression of the selected genes in wild-type and mutant strains, we performed RT-qPCR analyses. Results of this analysis showed a good correlation between protein-enrichment in OMVs and overexpression of the corresponding genes, suggesting that differences in OMV content between wild-type LVS and Δ*fupA/B* strains are linked to differences in gene expression (Table 1).

### FupA/B mutation in *F. tularensis* promotes biofilm formation

**Figure 6.**
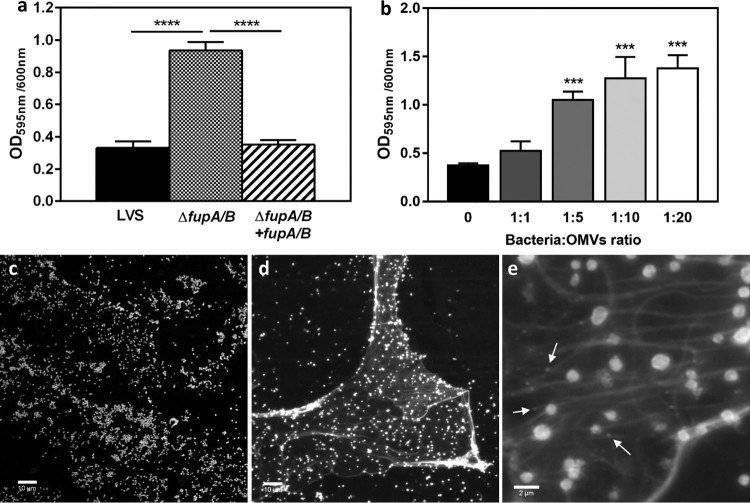
Biofilm formation by *F. tularensis* LVS. (a) Crystal violet staining was performed to assess the biofilm formation by *F. tularensis* LVS (black columns), LVSΔ*fupA/B* (dotted columns) and LVSΔ*fupA/B* complemented with *fupA/B* (hatched columns) grown for 72 h in 96 well plates at 37°C without shaking. (b) The biofilm formation was evaluated after 72 h incubation of *F. tularensis* LVS supplemented with different amount of freshly purified LVSΔ*fupA/B* OMVs numerated using the Nanosight Instrument and added in wells containing 2 × 10^8^ bacteria/ml at a final ratio bacteria:vesicles of 1:1–1:20. Data are expressed as mean ± SEM of 3 different experiments. ****P <* *0.0001, ***P <* *0.001; *P <* *0.05. (c) Biofilm formation was also examined using confocal laser-scanning microscopy from *F. tularensis* LVS and (d, e) LVSΔ*fupA/B* stained with FM®1-43X dye. Bar scales 10 µm (c, d) and 2 µm (e). Arrows indicate the presence of OMVs.

The increase in OMV secretion observed with the LVSΔ*fupA/*B strain may affect several important processes [27]. Indeed, these vesicles have been shown to promote bacterial resistance against antibiotics [27], a role which is of particular interest in the context of our work. OMVs exert this effect by acting as decoy targets for these compounds, ensuring the transfer of antibiotic-resistance genes or antibiotic-degrading enzymes, mediating drug export, and contributing to biofilm formation [27,37]. As putative structural components of the extracellular matrix, *Francisella* OMVs were thus proposed to contribute to biofilm formation. This protected mode of growth is associated with enhanced bacterial survival and persistence of *Francisella* spp. within the environment, but has never previously been implicated in antibiotic resistance [27,38]*.* Interestingly, results obtained here demonstrate that biofilm formation increased significantly with the FQ-resistant LVS strains lacking *fupA/B*. This effect was observed for both the genetically modified strain LVSΔ*fupA/B* (Figure 6(a)) and for the P12V3 isolate from the directed-evolution experiment (Supp Fig. 3). Importantly, we also noticed significantly enhanced biofilm production when the LVS cell suspension was supplemented with purified vesicles (Figure 6(b)).

**Figure 7.**
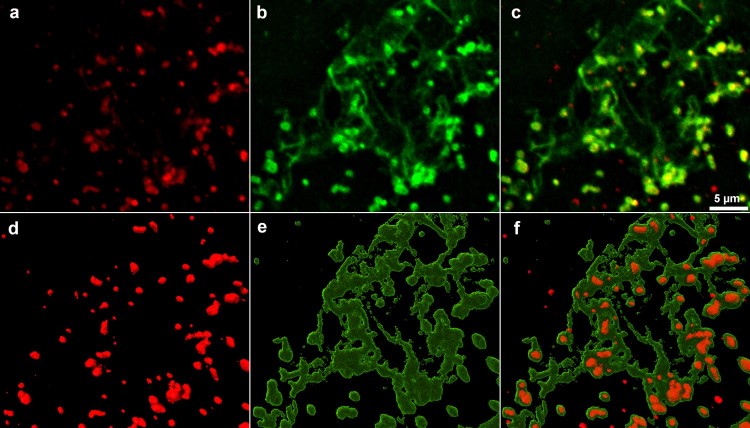
Cofocal laser-scanning microscopy images of *F. tularensis* LVSΔ*fupA/B*. Biofilms-embedded bacteria stained with (a) FM®1-43FX, (b) ConA-FITC, (c) merge. (d-f) 3D reconstruction of a, b and c. Each channel of the raw data (i. e, xyz files) were deconvoluted using the “iterative Deconvolve 3D” plugin (ImageJ software). The UCSF ChimeraX software [39] was used for 3D reconstruction of processed images.

Confocal laser-scanning microscopy (CLSM) analysis showed that, in contrast to LVS, LVSΔ*fupA/B* was embedded in an extracellular polymeric substance (EPS) (Figure 6(c-e)). The size of OMVs – around 200 nm – is close to the limit of resolution of confocal microscopy. As a result, their observation is tricky, but some particles nevertheless appear to be distinguished within the biofilm matrix (Figure 6(e), arrows). EPS production was further confirmed by specific staining (Figure 7). Interestingly, and in addition to the presence of α-mannopyranosyl and α-glucopyranosyl residues in EPS specifically stained with the fluorescent-lectin-concanavalin A, this analysis revealed that, when embedded in a three-dimensional matrix, the bacteria shift from a bacillus-coccobacillus planktonic form to a biofilm form characterized by the presence of clusters of bacterial cells (Figure 7; Supp Movie 2).

### Contribution of biofilm formation to FQ susceptibility

**Figure 8.**
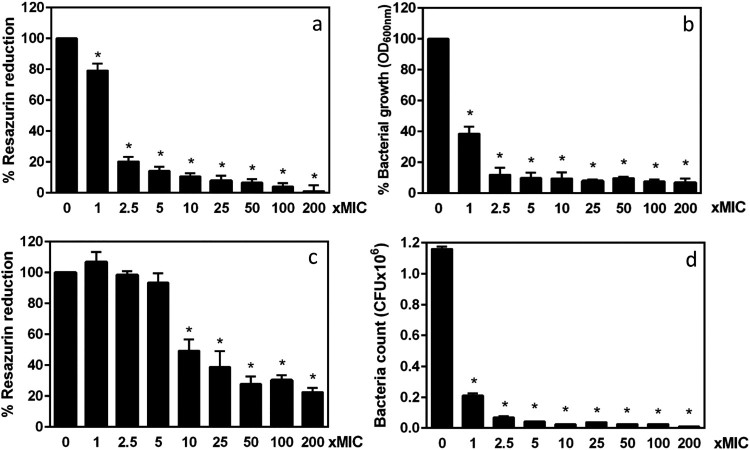
Comparative bactericidal effect of ciprofloxacin against planktonic and biofilm populations of *F. tularensis* LVSΔ*fupA/B.* The metabolic activity of planktonic (a) or biofilm bacteria (c) exposed for 24 h to increasing concentrations of ciprofloxacin was assessed by resazurin-reduction assay. The bacterial replication was monitored by OD_600nm_ value for planktonic cells (b) and by CFU counting for biofilm (d). Data represent the mean ± SEM of three different experiments performed in triplicate. *P <* *0.05.

To investigate whether the biofilm produced by LVSΔ*fupA/B* contributed to FQ resistance, planktonic and biofilm populations were exposed to increasing ciprofloxacin concentrations above the MIC. Bacterial survival was then determined by both the resazurin-based metabolic activity assays and CFU counting. Using the broth-dilution method and in line with the CLSI guidelines [24], the ciprofloxacin MIC was determined to be 0.064 µg/mL for LVSΔ*fupA/B* (Figure 2(b)). This value is considered a reference value for this type of experiment. The proportion of planktonic bacteria in each well after 72 h incubation under static conditions was measured to be 86.55% ± 0.85%, *n* = 58 (Supp Fig. 4) indicating that 13.45% ± 0.8%, *n* = 58, i.e. 1.8 × 10^8^ bacteria were possibly trapped into the biofilm matrix. Thus, and to adequately evaluate the efficiency of ciprofloxacin on biofilm *vs.* planktonic cells, we compared the metabolic activity of 72 h-biofilm cells with that of a bacterial cell suspension normalized for the CFU/mL concentration susceptible to be embedded in a 72 h-biofilm. Under these conditions, after incubation with 0.064 µg/mL of ciprofloxacin (MIC x1) for 24 h, the planktonic bacteria displayed a small but significant (*P *< 0.05) decrease in metabolic activity (Figure 8(a)) associated with decreased cell replication (Figure 8(b)). In biofilm conditions, LVSΔ*fupA/B* was much less vulnerable to ciprofloxacin, with alterations to metabolic activity detectable only from a ciprofloxacin concentration corresponding to 10x the MIC (Figure 8(c)). While the total biofilm biomass was harvested from the wells (as confirmed by crystal violet staining) the recovery of bacteria on PVX-CHA plates from untreated biofilm was lower than initially expected when taking in account the removal of bacteria during washing steps (around 3 × 10^7^ CFU/well). This could result from the formation of bacterial aggregates within the biofilm matrix that was only dissociated by pipetting without sonication or addition of detergent [40]. Interestingly, the growth defect of bacteria colonizing biofilm was found amplified upon antibiotic exposure. Thus, when biofilms were incubated for 24 h with a ciprofloxacin concentration of 0.16 µg/mL (MIC x2.5) the metabolic activity of bacteria was preserved while they lost their ability to grow on agar-enriched plates (Figure 8(d)). Importantly, we demonstrated the viability of LVSΔ*fupA/B* biofilm cells exposed to these ciprofloxacin conditions by using propidium iodide (PI) staining as a cell integrity indicator (Supp Fig. 5).

## Discussion

In this work we demonstrated that deletion of the hybrid gene *fupA/B* results in reduced FQ susceptibility in the *F. tularensis* LVS strain (Type B), and we characterized the phenotypic changes associated with and potentially explaining this antibiotic resistance, which was also observed following *fupA*, but not *fupB*, deletion in the highly virulent SCHU S4 strain (Type A). Although the role of the lipoproteins FupA and FupA/B in iron uptake and bacterial virulence has long been established [8,9], the results presented here are the first evidence of a role for these proteins in FQ resistance. The time-kill curves for FQ susceptibility generated in this study clearly show that LVSΔ*fupA/B* are FQ-tolerant bacteria, when compared to the wild-type strain. More strikingly, a similar approach revealed that LVSΔ*fupA/B* also exhibits enhanced survival when treated with the bactericidal antibiotic gentamicin. In contrast, no difference was observed with doxycycline, a result linked to the fact that tolerance applies only to bactericidal and not to bacteriostatic compounds [17]. The MDK metric used here thus allowed us to highlight a gentamicin tolerance phenotype which had been previously overlooked due to comparable MIC levels. This phenotype may well contribute to the relapses and therapeutic failures observed when dealing with tularemia patients. As a consequence, and as previously proposed [18], this method should be used as standard for the *in vitro* characterization of antibiotic-sensitivity in order to propose more appropriate treatments for recalcitrant infections.

Our data indicated that deletion of *fupA/B* results in a hypervesiculating phenotype in *Francisella* LVS. The formation of OMVs is a veritable bacterial secretion process, and has been described as a physiological response in Gram-negative bacteria exposed to various stresses [27]. In fact, and although the precise molecular mechanisms involved remain to be elucidated, it is now well-established that OMVs play a crucial role in protecting bacteria against antibiotics [41]. The capacity of several *Francisella* strains, including *F. tularensis* LVS and SCHU S4, to produce OMVs was previously reported [30,42–45], but the regulation of their biogenesis and secretion is still poorly documented. Three main mechanisms can trigger increased OMV budding: reduced lipoprotein-dependent cross-linking between the OM and the underlying peptidoglycan layer, accumulation of peptidoglycan exerting turgor pressure on the OM, and enrichment of the OM in phospholipids or LPS [46]. Several pieces of evidence obtained here suggest that the increased OMV secretion observed in LVSΔ*fupA/B* results from reduced envelope cross-linking caused by the lack of the FupA/B lipoprotein. First, the electrophoretic pattern of LPS for the different strains was unchanged, suggesting that the OMV overproduction is not induced by LPS remodeling. Secondly, the homogeneity of vesicle size indicates that the phospholipid composition which could affect the curvature is preserved whether FupA/B is expressed or not. Finally, our analyses revealed that whole cells and OMVs had similar LPS profiles, confirming the OM origin of vesicles. Another recently proposed mechanism for OMV biogenesis involves downregulation of a phospholipid transport system by Fur under iron-limiting conditions [47]. Although several combinatorial effects between iron and antibiotics have been described, an impressive heterogeneity in published results has been pointed out [48]. Indeed, we cannot formally exclude the hypothesis that the lower intracellular iron concentration detected in LVSΔ*fupA/B* in some way modulates the FQ resistance. It is interesting to note that the enhanced antibiotic resistance observed with iron-deficient bacteria is often associated with a growth defect [48]; no such growth restriction is observed upon deletion of *fupA/B*, which is not Fur-regulated.

The proteomic analysis presented here provides a unique repertoire of 801 proteins identified in OMVs purified from *F. tularensis* LVS. Many of the OMV-enriched proteins were OM and periplasmic proteins, a result which was expected given the vesicle biogenesis process [27], as discussed above. The extensive quantitative comparison of the protein content of OMVs derived from wild-type LVS and LVSΔ*fupA/B* revealed a subset of differentially-expressed proteins. With few exceptions, RT-qPCR showed the differentially-expressed targets to be in striking qualitative agreement with the differential abundance of the corresponding bacterial mRNAs. Thus, and as observed for LPS, the protein content of OMVs collected from bacteria grown up to the stationary phase under shaking probably reflects the underlying bacterial cell composition rather than resulting from selective sorting of cargo proteins [35]. Interestingly, the majority of changes arising from the *fupA/B* deletion was associated with upregulation of proteins involved in bacterial metabolism. It is now acknowledged that the metabolic alterations arising from antibiotic resistance do not necessarily reduce bacterial fitness, but can lead to a gain of fitness such that, in the absence of antibiotics, resistant populations grow as well as susceptible strains [49]. Enhanced metabolism could thus promote LVSΔ*fupA/B* replication and survival despite lower iron availability resulting from FupA/B deletion [8,9]. Alone, a reduced level of this essential element represents a major perturbation that most probably reduces LVSΔ*fupA/B* fitness that could be compensated for by metabolic adaptations such as those revealed by the results presented here. Another strategy displayed by this mutant which is also relevant for iron uptake is overexpression of the siderophore biosynthesis FslE protein encoded by FTL_1832. This protein presents some similarities with the cytosolic legiobactin A which has the capacity to stimulate the growth of iron-starved *Legionellae* [50]. The *fupA/B* deletion in *F. tularensis* LVS is also accompanied by an increase in RecA expression that could indirectly confer a phenotypic resistance and help bacteria to survive antibiotic stress [34]. In line with this hypothesis, we found that LVSΔ*fupA/B* was more tolerant to both ciprofloxacin and gentamicin than the wild-type strain.

Beyond the qualitative changes of the OMV composition that may reflect bacterial adaptation in response to antibiotic stress, we also observed a quantitative modulation of the OMV production rate*.* The hypersecretion of OMVs could be related to the formation of biofilm observed with LVSΔ*fupA/B*, a hypothesis supported by the capacity of purified vesicles to increase the amount of biofilm produced by these bacteria. The relationship between vesicles and biofilms, and more specifically a role for vesicles as part of the EPS has been described for several bacteria [51], but – although suggested [38] – never previously experimentally confirmed for *Francisella* spp. Through an accurate comparative analysis of the survival of planktonic and biofilm LVSΔ*fupA/B* exposed to FQ, we were able to demonstrate a clear relationship between the biofilm growth mode and reduced FQ susceptibility. Although biofilm bacteria from a number of species have been described as endowed with a greater capacity to resist antibiotics than their planktonic counterparts, a similar contribution to drug resistance was never previously examined before for *Francisella* spp. Interestingly, and although still metabolically active, our results indicate that biofilm-embedded LVSΔ*fupA/B* cells presented a growth defect and were likely to acquire a viable but non-culturable (VBNC) state, such as that described for *Staphylococcus aureus* [52]. We also noticed that LVSΔ*fupA/B* cells present within biofilms underwent morphological changes, shifting from a bacillus to a rounded shape, a phenomenon that may be related to a survival mode. A similar pattern was observed with *F. novicida* U112, an environmental strain endowed with a strong capacity to produce biofilm (paper submitted).

The topic of antibiotic tolerance is complex, and the resistance of *F. tularensis* to FQ is definitely not solely a consequence of DNA gyrase mutations [5,7]. The work presented in this paper reveals a new pathway based on FupA/B or FupA alterations through which FQ could drive the emergence of drug resistance. Data gathered here shed light on the tolerance phenotype observed for LVSΔ*fupA/B*, the increased MIC of biofilm bacteria as well as on their VBNC state, thus providing new opportunities for more comprehensive studies related to antibiotic challenge. Antibiotic tolerance/resistance is undoubtedly a multifactorial process, and we believe that these mechanisms contribute to treatment failures and to the development of persister cells responsible for relapse events in patients.

## Supplementary Material

Supplemental Material
