# Supplementary material for: Francisella tularensis: FupA mutation contributes to fluoroquinolone resistance by increasing vesicle secretion and biofilm formation
Source: Emerg Microbes Infect. 2019 Jun 4;8(1):808–22. doi: 10.1080/22221751.2019.1615848 (PMC6566608; doi:10.1080/22221751.2019.1615848)
Supplement: Supplemental Material [file TEMI_A_1615848_SM0550.zip › Supplementary Material/temi-2019-0044-20190503175547/doc/_TEMI_2019_0044_Renesto_SI_final_sup.docx]

Supplementary Information for

***Francisella tularensis*: FupA mutation contributes to fluoroquinolone resistance by increasing vesicle secretion and biofilm formation**

Claire Siebert^1,2^, Helena Lindgren^3^, Sabrina Ferré^4^, Corinne Villers^1,5^, Sandrine Boisset^1,2^,

Julien Perard^6^, Anders Sjöstedt^3^, Max Maurin^1,2^,

Céline Brochier-Armanet^7^, Yohann Couté^4^ and Patricia Renesto^1,*^

^1^TIMC-IMAG UMR 5525 - UGA CNRS, Grenoble Cedex 9, France;

^2^Centre Hospitalo-Universitaire Grenoble Alpes, Centre National de Référence des *Francisella*, Grenoble Cedex 9, France;

^3^Laboratory for Molecular Infection Medicine Sweden and Department of Clinical Microbiology, Umeå University, Umeå, Sweden;

^4^Université Grenoble Alpes, CEA, Inserm, IRIG-BGE, Grenoble, France;

^5^Université de Caen Normandie, EA4655 U2RM, Caen, France;

^6^Université Grenoble Alpes, CNRS, CEA, BIG-LCBM, Grenoble, France;

^7^Université Claude Bernard Lyon 1, CNRS, UMR5558, Laboratoire de Biométrie et Biologie Évolutive, Villeurbanne, France.

Corresponding author: Patricia Renesto

Email: [patricia.renesto@univ-grenoble-alpes.fr](mailto:patricia.renesto@univ-grenoble-alpes.fr)

**This PDF file includes:**

Supplementary Materials and Methods

Supplementary Figures 1 to 5

Supplementary Tables 1 and 3

Captions for movies S1 and S2

References for SI reference citations

**Other supplementary materials for this manuscript include the following:**

Movies 1 and 2

Supplementary Dataset 1

**Supplementary Materials and Methods**

**Bioinformatics analyses**

Complete bacterial proteomes, including 384 *Thiotrichales* proteomes, available at the NCBI (<https://www.ncbi.nlm.nih.gov/>) were surveyed using the BLASTP program (default parameters). FupA (FTT_0918), FupB (FTT_0919), FlsE (FTT_0025c), FmvA (FTT_0267), and FmvB (FTT_0602c) protein sequences from *F. tularensis* subsp. *tularensis* SCHU S4 were used as seeds, and identified 1,214 DUF3573-containing proteins. These proteins were exclusively present in *Thiotrichales* (*Gammaproteobacteria*) representatives (Supplementary dataset 1). The 384 *Thiotrichales* for which complete proteomes were available in the NCBI database include *Sulfurivirga*, *Thiomicrospira*, *Hydrogenovibrio*, *Cycloclasticus*, *Methylophaga*, *Allofrancisella*, *Beggiatoa*, *Caedibacter*, ‘*Candidatus* Thiomargarita’, *Fangia*, *Francisella*, *Piscirickettsia*, *Thioploca*, and *Thiotrix* genera.

Phylogenetic analysis was performed on a subsample of 62 DUF3573-containing proteins by retaining sequences from one representative strain per species or subspecies in the case of *F. tularensis*. The protein sequences were aligned using MAFFT, version 7.309 ^1, 2^, with the L-INS-i accurate option. The resulting multiple alignment was trimmed using BMGE version 1.12 ^3^, applying the BLOSUM45 substitution matrix. A maximum likelihood tree was inferred using IQ-TREE version 1.5.3 ^4^ with the LG+Г4 evolutionary model, which was the best model identified by IQ-TREE according to the Bayesian Information Criterion. The robustness of the branches on the inferred tree was assessed by applying the ultrafast bootstrap approximation approach implemented in IQ-TREE (1,000 replicates). The figure displaying the tree was drawn with iTOL version 3 ^5^.

**Construction of the FTL_0439 knock-out strain**

The recombinant plasmid pMP812-Δ*fupA/B* was obtained by overlap PCR. A 829 bp 5’ flanking region and a 421 bp intragenic region of *fupA/B* were amplified from LVS genomic DNA and using primers p7/p8 and p9/p10, respectively (Supp Table 3). Primers p8 and p9 have an overlapping sequence of 13 nucleotides resulting in deletion of the *fupA/B* coding sequence from nucleotides 1 to 1,232 after cross-over PCR with primers p7 and p10 containing specific sequences of restriction sites NotI and BamHI to limit the fusion. The final 1,261 bp PCR product was digested by NotI and BamHI and cloned into the digested pMP812 to generate pMP812-Δ*fupA/B*. This suicide plasmid was introduced into *F. tularensis* LVS strain by electroporation and recombinant cells which have integrated the plasmid into their genomes, were selected on Polyvitex-enriched chocolate agar (PVX-CHA) plates (bioMérieux, Marcy l'Étoile, France) supplemented with kanamycin (10 µg/mL). Plasmid integration was checked on isolated colonies using primers located on the pMP812 and either downstream or upstream the integration region (primer pairs p11/p12, p13/p14). Positive clones were then grown in liquid medium without selection to allow the second recombination to occur and were plated on PVX-CHA plates supplemented 5% (w/v) sucrose. Colonies were analyzed for FTL_0439 deletion with primers p11/p14. The gene deletion was confirmed through PCR and sequence analysis.

**Minimal Inhibitory Concentration Measurement (MIC)**

For the *F. tularensis* LVS strains, the MICs were determined using a broth micro dilution method recommended by the Clinical Laboratory Standards Institute (CLSI), as previously described ^6^. To maintain the plasmids into the evolution strains and LVSΔ*fupA/B* transformed with pMP818 or pMP828-*fupA/B,* kanamycin (10 µg/mL) was added to the Mueller-Hinton broth supplemented with 2% PolyViteX® (MH-PVX) (bioMérieux, Marcy L'Etoile, France). One row of a 96-well microtiter plate was filled with 75 μL of two-fold serial dilutions of ciprofloxacin in MH-PVX medium, to obtain final FQ concentrations ranging from 0.125 to 32 μg/mL for the evolution mutants and from 0.001 to 0.5 µg/mL for the other strains. A bacterial inoculum (75 μL per well, 5 × 10^5^ colony-forming unit (CFU)/mL of final inoculum) was then added to each well. Antibiotic free cultures were used as a positive control and MH-PVX medium served as a negative control. Microplates were incubated at 37°C in a 5% CO_2_ atmosphere. The MICs were read after 48 h culture incubation. MICs corresponded to the minimum ciprofloxacin concentration that allowed complete inhibition of visual growth of bacteria. Experiments were conducted at least thrice in triplicate.

The *F. tularensis* SCHU S4, SCHU S4Δ*fupA*, SCHU S4Δ*fupB* and SCHU S4Δ*fupA*Δ*fupB* strains were exposed to a two-fold serial dilutions of ciprofloxacin in Chamberlain`s medium in a range from 0.016 to 0.5 μg/mL. The OD_600nm_ of the inoculum was 0.02. The MICs were read after 72h and corresponded to the minimum ciprofloxacin concentration where the OD_600nm_ from triplicate cultures did not significantly exceed 0.02 (*P* > 0.05). Otherwise the MIC assay of ciprofloxacin for the SCHU S4 strain and mutants was determined essentially as described for the LVS strain and mutants.

**Production of anti-FupA antibody**

All primers used in cloning and sequencing steps are listed in Supp Table 3. Gene-specific primers p1/p2 and the High-Fidelity PCR master mix (Phusion, Finnzymes) were used to amplify the N-terminal end of *fupA/B* (pb 4 to 528) from *F. tularensis* LVS genomic DNA. The resulting PCR product was cloned into the pDEST-17 vector, which contains a 6xHis N-terminal tag sequence, using the Gateway cloning system (Invitrogen, Carlsbad, CA) according to the manufacturer’s instructions. Construct integrity was confirmed by DNA sequencing (Eurofins, Ebersberg, Germany).

E. coli, BL21(DE3) strain, was used as the host for protein expression. The cells were grown in Luria broth (LB) supplemented with ampicillin (100 µg/mL) until the culture suspension reached an absorbance of approximately 0.6. Protein expression was induced by addition of 0.5 mM isopropyl-β-D-thiogalactopyranoside, and overnight incubation at 16 °C. Cells were pelleted by centrifugation (5,000 x *g*, 20 min), resuspended in lysis buffer (50 mM Tris pH 8.8, 200 mM NaCl, 10 mM imidazole, 1% CHAPS and Complete Protease Inhibitor® (Roche Diagnostics)), then disrupted by sonication. After centrifugation (20,000 rpm, 30 min, 4 °C) the bacterial lysate was affinity-purified on a Ni^2+^-NTA column (Qiagen). Following extensive washing (50 mM Tris pH 8.8, 200 mM NaCl, 20 mM imidazole), the protein was eluted with 300 mM imidazole. Eluted fractions were analyzed on 12% SDS-PAGE and visualized by Coomassie blue staining. The fractions containing the purified protein were pooled and concentrated using an Amicon Ultra centrifugation device with a 10-kDa cut-off, and further purified by size exclusion chromatography on an ENrich SEC 650 column in the NGC Chromatography System (Biorad). The purity of the N-ter-FupA/B protein was assessed by SDS-PAGE. The protein was concentrated to 1 mg/mL for use in rabbit immunization protocols to produce a polyclonal antibody (Biotem, France).

**Measuring iron content**

Intracellular iron concentrations were measured on stationary phase bacteria grown for 15 h in a shaking incubator (200 rpm at 37 °C) in modified Mueller Hinton medium without iron supplementation. Cells were then washed several time with PBS-EDTA 10 mM before hydrolysis with HNO_3_ at 65% overnight at 95 °C, and Inductively Coupled Plasma-Atomic Emission Spectroscopy (ICP-AES) (Shimadzu ICP 9000 with Mini plasma Torch in axial reading mode) measurements were performed as previously described ^7^. Ytterbium was used as an internal standard to prevent calibration drift and fluidic perturbation. Results were expressed in µg/L.

# Western blot analysis of FupA/B expression.

# Bacterial pellets resuspended in Laemmli loading buffer supplemented with NP-40 (1% final concentration) were subjected to SDS-PAGE on a 12% polyacrylamide gel. Proteins were transferred onto a nitrocellulose membrane (Trans-Blot Turbo, Biorad). Western blot analysis was performed by a standard procedure using a polyclonal antibody to FupA/B (1:40,000) and a peroxidase-conjugated rabbit secondary antibody (1:10,000; Jackson ImmunoResearch, Baltimore, PA, USA). The mouse anti-*Francisella*-IglC (1:2000; *bei* Resources, Manassas, VA, USA) and secondary peroxidase-conjugated anti-mouse (1:10,000; Jackson ImmunoResearch, Baltimore, PA, USA) combination was used as a positive control. Antibody binding was revealed by enhanced chemiluminescence (Clarity^TM^ Western ECL, Biorad®); signal was detected and analyzed using the Biorad Chemidoc XRS + System.

# Western blot analysis of LPS expression

# LPS was detected as described above starting from normalized quantities of bacterial lysates or OMVs which were separated on 4-20% gradient SDS-PAGE gels (Biorad)*.* The polyclonal rabbit anti-*F. tularensis* LVS Lipopolysaccharide (LPS) from Kerafast (Kerafast, Inc., Boston, MA, USA) was diluted 1:10,000 and the HRP-conjugated rabbit secondary antibody was used at 1:10,000.

**Dynamic Light Scattering**

Dynamic Light Scattering (DLS) (Wyatt instrument) was used to measure size, size-distribution and polydispersity of purified OMV particles in-solution. Calibration was performed using 50 nm and 100 nm latex beads (Malvern). Samples were analyzed in duplicate, with each measure repeated 10 times. Data were merged to estimate the hydrodynamic radius of OMV particles. For all samples analyzed, the polydispersity index was less than 0.16.

**Mass spectrometry-based quantitative proteomics analyses**

OMV proteins were stacked in the top of an SDS-PAGE gel (4-12% NuPAGE, Life Technologies) and revealed with Coomassie blue R-250 before in-gel digestion using modified trypsin (Promega, sequencing grade), as previously described ^8^. The resulting peptides were analyzed by online nanoliquid chromatography coupled to tandem MS (UltiMate 3000 and LTQ-Orbitrap Velos Pro, Thermo Scientific). Peptides were sampled on a 300 µm x 5 mm PepMap C18 precolumn and separated on a 75 µm x 250 mm C18 column (PepMap, Thermo Scientific) using a 120-min gradient. MS and MS/MS data were acquired using Xcalibur (Thermo Scientific). Peptides and proteins were identified and quantified in MaxQuant (version 1.5.8.3) ^9^ using the *F. tularensis subsp. holarctica* LVS database from MicroScope ^10^ and the frequently observed contaminant database included in MaxQuant. Trypsin was selected as the enzyme, and up to two missed cleavages were allowed. Peptide modifications allowed during the search were as follows: carbamidomethylation (C, fixed), acetyl (Protein N-ter, variable) and oxidation (M, variable). Minimum peptide length was set to 7 amino acids. Minimum number of peptides and razor + unique peptides were both set to 1. Maximum false discovery rates - calculated by a reverse database strategy - were set to 0.01 at peptide and protein levels. The mass spectrometry proteomics data have been deposited to the ProteomeXchange Consortium via the PRIDE ^11^ partner repository under dataset identifier PXD010305.

Statistical analyses were performed in ProStaR ^12^. Proteins identified in both the reverse and contaminant databases, proteins only identified by site, proteins identified by a single peptide and proteins exhibiting fewer than 3 iBAQ values in a single condition were discarded from the list. After log2 transformation, iBAQ values were normalized by median centering before imputing missing values (replacing missing values by the 1 percentile value for each column); statistical testing was conducted by applying a limma *t*-test. Differentially-expressed proteins were sorted out using a log2 (fold change) cut-off of 2 and a p-value cut-off of 0.01 (FDR less than 5% according to the Benjamini-Hochberg method).

Subcellular localization of proteins from the *F. tularensis subsp. holarctica* LVS database were predicted using PSORTb (version 3.0.2, ^13^). Lipoprotein signal peptides, other signal peptides and N-terminal membrane helices in proteins of the *F. tularensis subsp. holarctica* LVS database were predicted using LipoP ^14^. If several prediction categories (excluding cytoplasmic) emerged for the same protein, a delta score was calculated; if this delta score was less than 5, both predictions were retained.

# Quantitative real-time PCR

# Gene transcription levels were determined by quantitative Real-Time (RT-qPCR) performed on 500-µL bacterial cultures grown to stationary phase (24 h). Total RNA was extracted from bacterial pellets resuspended in RNAprotect Bacteria Reagent (Qiagen, USA) and using the RNeasy Mini Kit (Qiagen) according to the manufacturer’s instructions. Contaminating genomic DNA was removed using the Turbo DNA-free^TM^ kit (Ambion). The final RNA concentration and purity were measured on a Nanodrop spectrophotometer (Nanodrop Technologies Inc.). Reverse transcription was performed with 1 µg of RNA using the iScript^TM^ Reverse Transcription Supermix (Biorad). The product was used as template for qPCR. For each extraction, a control reaction without reverse transcriptase was used to verify the absence of genomic DNA contamination of RNA samples. Quantitative PCR was performed on a StepOnePlus Real-Time PCR System (Applied Biosystems) in 10 μL reaction volume containing 1 μL of template and 9 µL of fast SYBR® green PCR Master Mix (Thermo Fisher Scientific) containing 300 nM of gene-specific primers (Supp Table 3). Relative changes in gene expression between LVS and LVSΔ*fupA/B* were assessed by applying the delta-delta cycle threshold (Ct) method and using rRNA 16S as reference gene. All samples and controls were run in duplicate with at least two different batches of cDNA.

**CFU counting**

Biofilms were disrupted by vigorous pipetting in PBS, and the resulting cell suspensions were serially diluted in PBS. 100 μL of at least four different dilutions for each well were plated on PVX-CHA plates. CFU counts were determined after incubation at 37 °C under 5% CO_2_ for 48 h.

**Fluorescence microscopy**

Bacteria were grown as described above in conditions allowing biofilm formation. For each time point (t_0_, 24 h, 48 h and 72 h), samples collected at the air-water interface were fixed with 4% (w/v) paraformaldehyde for 10 min at room temperature and washed twice with sterile PBS. Slides were then incubated with FM^®^1-43FX membrane probe (Life Technologies) for 30 min at room temperature and mounted with Vectashield mounting medium (Vector Laboratories, California, USA). Bacteria and biofilms were observed and imaged with a confocal laser-scanning microscope (LSM710 Zeiss) equipped with a 63x/1.4 oil-immersion objective. Images were acquired using the Zeiss Zen software. For 3D reconstructions, the raw data (i.e., xyz files) were processed with ImageJ software to crop the sequence and region of interest; the z stack from each channel was deconvoluted using the “Iterative Deconvolve 3D” plugin with a z-step of 0.3 μm. UCSF ChimeraX software ^15^ was used for 3D reconstruction and to produce movies from processed images.

**Supplementary Figure 1**

Alignment of the *F. tularensis* LVS (WP_011457377.1) FupA/B sequence with the *F. tularensis* SCHU S4 sequences from (**a**) FupA (YP_169915.1) and (**b**) FupB (YP_169916.1). Alignments were produced using ClustalX **^16^**. The figure was generated using Espript3 **^17^**. Identical residues are colored in red and blue boxes represent homologous residues. The arrow corresponds to the lipoprotein site.

**
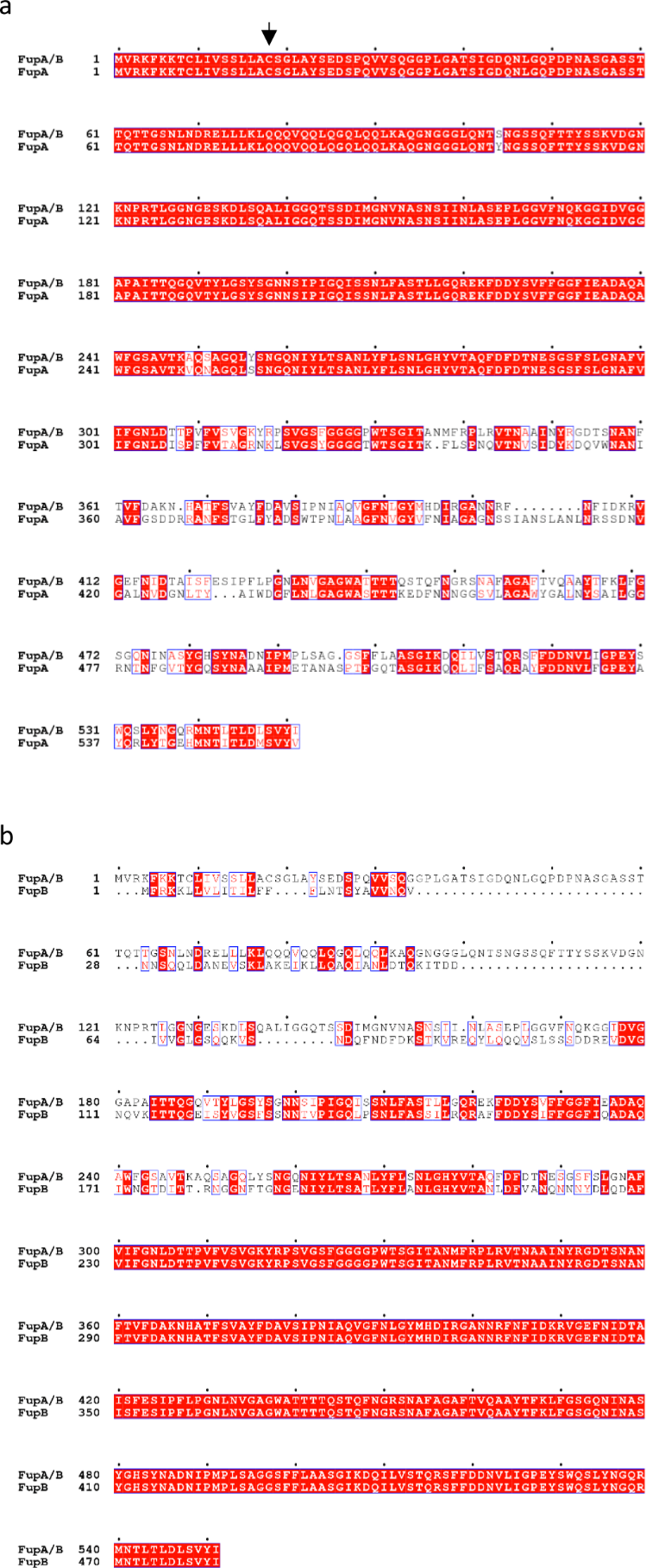
**

**Supplementary Figure 2 : Cloning and phenotypic analysis of LVSΔ*fupA/B.***

(**a**) LVS transformed with pMP812-Δ*fupA/B* and grown on chocolate agar supplemented with sucrose produce both large (1, 2) and small (3, 4, 6) colonies. (**b**) PCR screen of selected colonies using primers flanking *fupA/B* and genomic DNA from LVS as control. Samples lacking *fupA/B* (3, 4, 6) correspond to bacteria displaying a small-colony phenotype. No amplification was obtained from sample 5 (**c**) Confirmation of *fupA/B* deletion by western blot analysis of whole bacterial extracts. Anti-IglC was used as positive control. (**d**) Growth curves for LVS and LVSΔ*fupA/B* at 37 °C under shaking in MMH supplemented with iron and glucose. (**e**) Iron concentration measured by ICP-AES. Sucrose-resistant colonies lacking *fupA/B* displayed a small-colony phenotype, but their growth kinetics in liquid MMH medium were comparable to those of wild-type LVS.


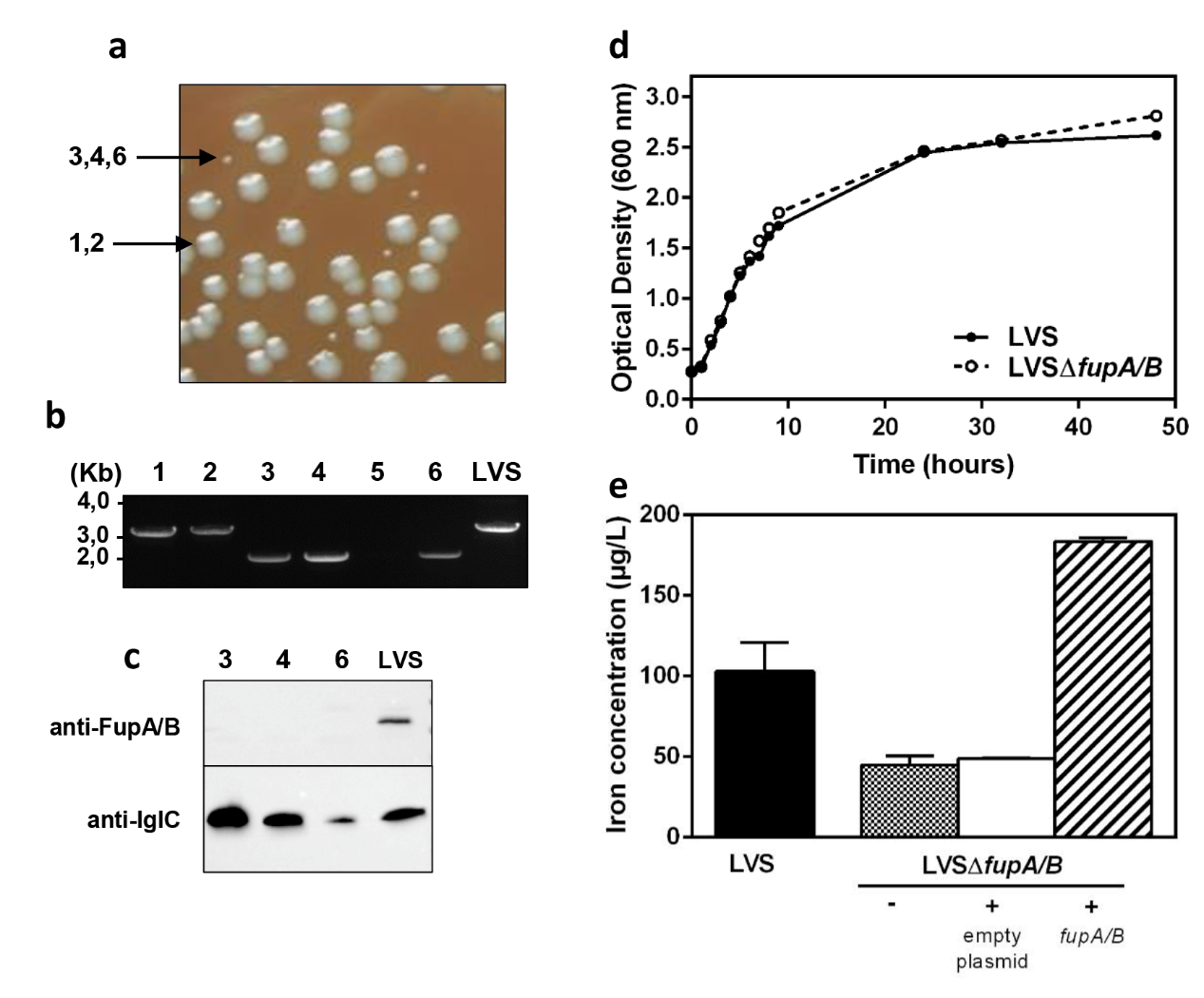


**Supplementary Figure 3 :** Crystal violet measurement of the biofilm formed by LVS mutants obtained from the directed-evolution experiment ^6^ showing that the mutant P12V3 lacking *fupA/B* produced more biofilm than the isolate P2V1, an effect be restored by gene complementation. *** *P* < 0.005.





**Supplementary Figure 4 : Proportion of planktonic bacteria after 72 h growth under static conditions.** After 72 h incubation at 37 °C under static conditions, the OD_600nm_ of the microtiter plate inoculated with 2x10^8^ bacteria/well and containing both planktonic and biofilm (TOTAL) bacteria was measured. The OD_600nm_ corresponding to planktonic (PK) bacteria was determined after transfer of the liquid fraction to another microtiter plate. Data indicated that the majority of bacteria (86.55% ± 0.85%, n=58) remains planktonic.

**
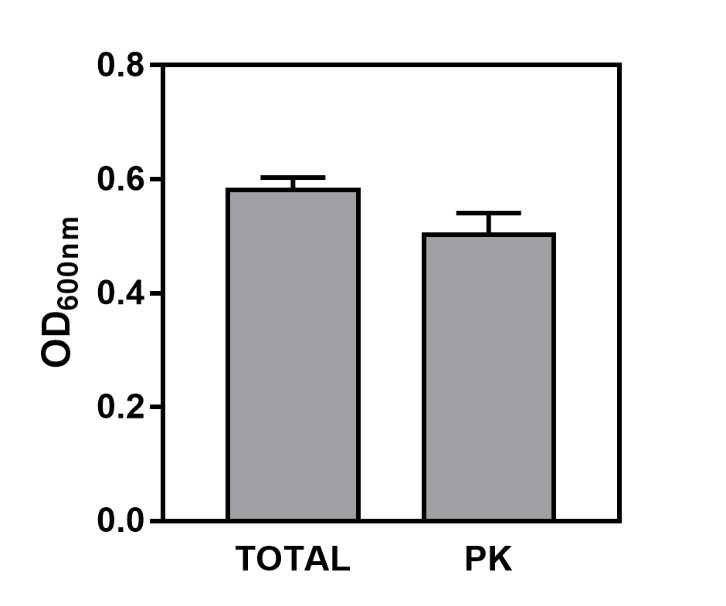
**

**Supplementary Figure 5 : Ciprofloxacin does not alter bacterial viability, as measured by propidium iodide (PI) staining.**

LVSΔ*fupA/B* biofilms were incubated with ciprofloxacin (MIC x2.5, 24 h) or H_2_O_2_ **(**100 mM, 30 min) before staining with PI. Results were expressed as the ratio between fluorescence values measured for biofilms exposed to various treatments and fluorescence values measured for samples permeabilized with paraformaldehyde before staining.

In contrast to what was observed after treatment with H_2_O_2_, no lytic cell death was detected upon exposure to ciprofloxacin. The results presented are representative of two independent experiments with 3 to 6 replicates each. * *P* < 0.05. *NS* not significant.

**
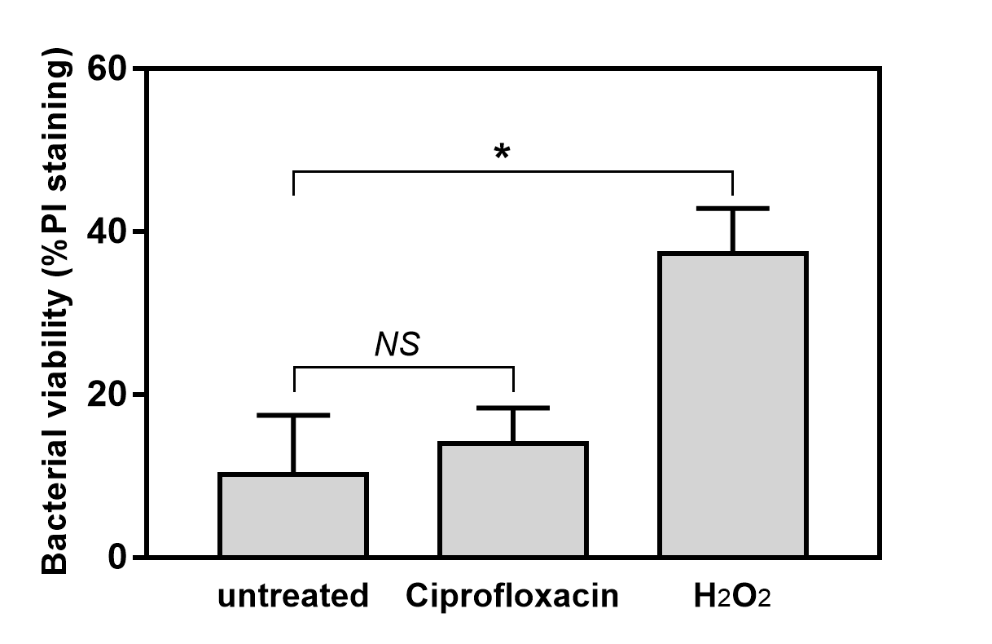
**

**Supplementary Table 1. Features of paralogous FupA proteins.**

Genes encoding the FupA paralogs in the different *Francisella* strains alongside their specific protein features: functional domains, signal peptides, transmembrane domains (TM) according to InterPro, SignalP, TMHMM, and Prosite. The prokaryotic membrane lipoprotein lipid attachment site (PROKAR_LIPOPROTEIN) corresponds to residue C18 on FupA and FupA/B. The cysteine protease inhibitor signature (CYSTATIN) was located in the AA 506-519 region in FupA. When present, the position of the TM domain is indicated.

* The FTT_0267 sequence lacks the first 150 AA found in other FmvA proteins. As a result, no TM domain is detected in this protein.

| Protein  name | *F. tularensis* SCHU S4 | *F. tularensis*  LVS | *F. novicida* | DUF3573 | Signal peptide | Transmembrane domain | PROKAR  lipoprotein | CYSTATIN |
| --- | --- | --- | --- | --- | --- | --- | --- | --- |
| FupA | FTT_0918 (557 AA) | - | FTN_0444 (557 AA) | x | x | - | x | x |
| FupB | FTT_0919 (481 AA) | - | FTN_0445 (481 AA) | x | - | Single TM | - | - |
| FupA/B | - | FTL_0439 (551 AA) | - | x | x | - | x | - |
| FmvA | FTT_0267* (372 AA) | FTL_0147 (512 AA) | FTN_0142 (513 AA) | x | - | Single TM* | - | - |
| FmvB | FTT_0602c (492 AA) | FTL_0867 (485 AA) | - | x | -/+ | - | - | - |
| FslE | FTT_0025c (509 AA) | FTL_1836 (509 AA) | FTN_1686 (509 AA) | x | - | Single TM | - | - |

**Supplementary Table 3. Primers used in this study.**

| Primer name | 5’-3’ sequences | Specific features (bold) |
| --- | --- | --- |
| ***FupA/B cloning and expression*** | | |
| p1 | gggg**acaagtttgtacaaaaaagcaggc**ttagaaaacctgtacttccagggtgtgcgtaaatttaaaaaaacctg | attB1 site |
| p2 | gggg**accactttgtacaagaaagctgggtc**ttattagataccgcctttttggttaaagacgcc | attB2 site |
| ***Complementation plasmid construction*** | | |
| p3  p4  p5  p6 | gtgaaaa**ggatcc**cgcaataatcactatccagaag  catttta**tccgga**ctacatgataatgataacgaatatc  caacaag**tccgga**tttggtttgccaattttttattag  cctaa**gaattc**ctctaaatatgtctagatataaactg | BamHI site  BspEI site  BspEI site  EcoRI site |
| ***Suicide plasmid construction*** | | |
| p7  p8  p9  p10 | aattaa**gcggccgc**tagtgctggttgcggtatcgc  **ttaaattggcata**tatggtgcatacgtcgg  **tatgccaatttaa**tggagaatttaatatcgataccgc  aattaa**ggatcc**gatataaactgaaagatctaatg | NotI site  Overlap with p9  Overlap with p8  BamHI site |
| ***Plasmid screening*** | | |
| p11 | gctgcaagtggtatagatc |  |
| p12 | gctcgaaattaaccctcac |  |
| p13 | tattaccgcctttgagtgagc |  |
| p14 | taatacttcttaactagttacttcc |  |
| ***qRT-PCR*** |  |  |
| gene | Forward (F) and reverse (R) primers | Amplicon (nt) |
| FTL_1931 | FTL_1931_196F TTATTACAGCATCATCGTATGG  FTL_1931_358R TACCAATCCCCTCGATAAGC | 163 |
| FTL_1836 | FTL_1836_F GGTGGTGCACCTGCTATTACAACCC  FTL_1836_R CCTTAACCCTATTACAGTTGAGGC | 130 |
| FTL_1835 | FTL_1835_54F TGGTTTTTAGGTAATATGGGCG  FTL_1835_215R TAGATTTCAGATAGTGGCCC | 162 |
| FTL_1842 | FTL_1842_86F TTAAAGAGCAAGATAAGCGC  FTL_1842_254R ACATCTTAGAGGCAGCCG | 186 |
| FTL_0146 | FTL_0146__F CTATTAGGTAAGTCCGGCTCAGG  FTL_0146__R GTAAAACTGTTAACCATGGCATCAGTGC | 172 |
| FTL_0012 | FTL_0012_78F AGGAGATCAAGAAGCTGC  FTL_0012_242R TACCGCCTTGCTTTTGGC | 182 |
| FTL_1644 | FTL_1644_F TGGAAATATGGGGTACTCAAAGTGG  FTL_1644_R AAACTACAACTGTCTCGCGCTG | 117 |
| FTL_1850 | FTL_1850_F TACAGCTGCGGAAATTCGTGCTAG  FTL_1850_R CCAGTTTTAGCTGTAAATTGCTCCG | 129 |
| FTL_1274 | FTL_1274_F CGGCTATTCAAAATCAAGTTCGCC  FTL_1274_R CCAACGGTTCAGATAAATCACGAACTC | 125 |
| FTL_0203 | FTL_0203_F CACAAACTGCTATTGGTGATGCTATAGG  FTL_0203_R TGCCTGTAGTGGCTGTAGTGTCCC | 131 |
| FTL_1724 | FTL_1724_F GGCTTATGCTCTAGCGATTACTAATGGC  FTL_1724_R GGAGCCAAAAATAACCGCGTTTG | 109 |
| FTL_1025 | FTL_1025_F GTTTGCCGTTTCACTATAGAAGGTGTG  FTL_1025_R CCAGTTACGCGACTTGGTACGATT | 110 |
| FTL_1045 | FTL_1045_F GGCTCTACCTTTTGGTATAAAGCCAAA  FTL_1045_R TTATGATCGCTGGGGAATTCCG | 124 |
| FTL_1407 | FTL_1407_F GTGGTTCCTAAAGCTGAAGCAATTGAG  FTL_1407_R GGGTATATATTTTCATCTGCTCATCAGC | 106 |
| FTL_0449 | FTL_0449_F GCTCAGCGAGAGAGATCTTATTAAAATG  FTL_0449_R CCCAAAACTTTTTCAGCATCTTGG | 93 |
| FTL_1591 | FTL_1591_F GCAGTTTGTATAGGTCCTCCTGCTCC  FTL_1591_R AACCATAACCTGGATGTATCGCATCAG | 109 |
| FTL_1899 | FTL_1899_F GTTCAGATCCTGTGCCTCATCTTAGATC  FTL_1899_R TCTCCTGTAGCTTGGTTTGTTGAAGAAC | 124 |
| FTL_0738 | FTL_0738_F GCATGCTTAATCACCCTACTTTTTTCC  FTL_0738_R TGGCATACCAAACTCTTGCATATATCC | 114 |
| FTL_1832 | FTL_1832_F TTGGCAATGGTTAACTACAACGATGTC  FTL_1832_R CCTATTTTGGTTTTTGAACTGGGGTG | 108 |
| FTL_0003 | FTL_0003_436_F AAGCCTCAAGGTTAATAGCC  FTL_0003_593_R TTTCACATCTGACTTAACAAACC | 158 |

Supplementary Movie 1. Nanoparticle tracking of purified OMVs reveals a homogeneous suspension. Nanoparticle tracking analysis (Nanosight, Malvern Instrument) was performed on vesicles purified from LVSΔ*fupA/B*. Image size recorded by video was approximately 100 μm × 80 μm.

**Supplementary Movie 2.** Three-dimensional visualization of LVSΔ*fupA/B* biofilm.

Biofilm-residing LVSΔ*fupA/B* embedded in an extracellular matrix. Bacteria were stained with FM^®^1-43FX membrane probe and EPS with concanavalinA-FITC.

Additional data – Dataset S1 (separate file)

Sequence data used to infer the phylogenetic tree presented in Fig. 1.

Additional data – Supplementary Table 2 (separate file)

List of proteins identified by proteomics in OMVs from wild-type LVS and LVSΔ*fupA/B*.

**References**

1. Katoh, K., Misawa, K., Kuma, K. & Miyata, T. MAFFT: a novel method for rapid multiple sequence alignment based on fast Fourier transform. *Nucleic Acids Res* **30**, 3059-3066 (2002).

2. Katoh, K. & Standley, D.M. MAFFT multiple sequence alignment software version 7: improvements in performance and usability. *Mol Biol Evol* **30**, 772-780 (2013).

3. Criscuolo, A. & Gribaldo, S. BMGE (Block Mapping and Gathering with Entropy): a new software for selection of phylogenetic informative regions from multiple sequence alignments. *BMC Evol Biol* **10**, 210 (2010).

4. Nguyen, L.T., Schmidt, H.A., von Haeseler, A. & Minh, B.Q. IQ-TREE: a fast and effective stochastic algorithm for estimating maximum-likelihood phylogenies. *Mol Biol Evol* **32**, 268-274 (2015).

5. Letunic, I. & Bork, P. Interactive tree of life (iTOL) v3: an online tool for the display and annotation of phylogenetic and other trees. *Nucleic Acids Res* **44**, W242-245 (2016).

6. Sutera, V., Levert, M., Burmeister, W.P., Schneider, D. & Maurin, M. Evolution toward high-level fluoroquinolone resistance in Francisella species. *J Antimicrob Chemother* **69**, 101-110 (2014).

7. Perard, J. *et al.* Quaternary Structure of Fur Proteins, a New Subfamily of Tetrameric Proteins. *Biochemistry* **55**, 1503-1515 (2016).

8. Casabona, M.G., Vandenbrouck, Y., Attree, I. & Coute, Y. Proteomic characterization of Pseudomonas aeruginosa PAO1 inner membrane. *Proteomics* **13**, 2419-2423 (2013).

9. Cox, J. & Mann, M. MaxQuant enables high peptide identification rates, individualized p.p.b.-range mass accuracies and proteome-wide protein quantification. *Nat Biotechnol* **26**, 1367-1372 (2008).

10. Medigue, C. *et al.* MicroScope-an integrated resource for community expertise of gene functions and comparative analysis of microbial genomic and metabolic data. *Brief Bioinform* (2017).

11. Vizcaino, J.A. *et al.* 2016 update of the PRIDE database and its related tools. *Nucleic Acids Res* **44**, D447-456 (2016).

12. Wieczorek, S. *et al.* DAPAR & ProStaR: software to perform statistical analyses in quantitative discovery proteomics. *Bioinformatics* **33**, 135-136 (2017).

13. Yu, N.Y. *et al.* PSORTb 3.0: improved protein subcellular localization prediction with refined localization subcategories and predictive capabilities for all prokaryotes. *Bioinformatics* **26**, 1608-1615 (2010).

14. Juncker, A.S. *et al.* Prediction of lipoprotein signal peptides in Gram-negative bacteria. *Protein Sci* **12**, 1652-1662 (2003).

15. Goddard, T.D. *et al.* UCSF ChimeraX: Meeting modern challenges in visualization and analysis. *Protein Sci* **27**, 14-25 (2018).

16. McWilliam, H. *et al.* Analysis Tool Web Services from the EMBL-EBI. *Nucleic Acids Res* **41**, W597-600 (2013).

17. Robert, X. & Gouet, P. Deciphering key features in protein structures with the new ENDscript server. *Nucleic Acids Res* **42**, W320-324 (2014).
